# The developmental genetic architecture of vocabulary skills during the first three years of life: Capturing emerging associations with later-life reading and cognition

**DOI:** 10.1371/journal.pgen.1009144

**Published:** 2021-02-12

**Authors:** Ellen Verhoef, Chin Yang Shapland, Simon E. Fisher, Philip S. Dale, Beate St Pourcain

**Affiliations:** 1 Language and Genetics Department, Max Planck Institute for Psycholinguistics, Nijmegen, The Netherlands; 2 International Max Planck Research School for Language Sciences, Nijmegen, The Netherlands; 3 MRC Integrative Epidemiology Unit, University of Bristol, Bristol, United Kingdom; 4 Population Health Sciences, University of Bristol, Bristol, United Kingdom; 5 Donders Institute for Brain, Cognition and Behaviour, Radboud University, Nijmegen, The Netherlands; 6 Speech & Hearing Sciences, University of New Mexico, Albuquerque, New Mexico, United States of America; Newcastle University, UNITED KINGDOM

## Abstract

Individual differences in early-life vocabulary measures are heritable and associated with subsequent reading and cognitive abilities, although the underlying mechanisms are little understood. Here, we (i) investigate the developmental genetic architecture of expressive and receptive vocabulary in early-life and (ii) assess timing of emerging genetic associations with mid-childhood verbal and non-verbal skills. We studied longitudinally assessed early-life vocabulary measures (15–38 months) and later-life verbal and non-verbal skills (7–8 years) in up to 6,524 unrelated children from the population-based Avon Longitudinal Study of Parents and Children (ALSPAC) cohort. We dissected the phenotypic variance of rank-transformed scores into genetic and residual components by fitting multivariate structural equation models to genome-wide genetic-relationship matrices. Our findings show that the genetic architecture of early-life vocabulary involves multiple distinct genetic factors. Two of these genetic factors are developmentally stable and also contribute to genetic variation in mid-childhood skills: One genetic factor emerging with expressive vocabulary at 24 months (path coefficient: 0.32(SE = 0.06)) was also related to later-life reading (path coefficient: 0.25(SE = 0.12)) and verbal intelligence (path coefficient: 0.42(SE = 0.13)), explaining up to 17.9% of the phenotypic variation. A second, independent genetic factor emerging with receptive vocabulary at 38 months (path coefficient: 0.15(SE = 0.07)), was more generally linked to verbal and non-verbal cognitive abilities in mid-childhood (reading path coefficient: 0.57(SE = 0.07); verbal intelligence path coefficient: 0.60(0.10); performance intelligence path coefficient: 0.50(SE = 0.08)), accounting for up to 36.1% of the phenotypic variation and the majority of genetic variance in these later-life traits (≥66.4%). Thus, the genetic foundations of mid-childhood reading and cognitive abilities are diverse. They involve at least two independent genetic factors that emerge at different developmental stages during early language development and may implicate differences in cognitive processes that are already detectable during toddlerhood.

## Author summary

Differences in the number of words young children produce (expressive vocabulary) and understand (receptive vocabulary) can be partially explained by common genetic variation, and are related to reading and cognitive abilities later in life. Here, we studied genetic influences underlying expressive and receptive vocabulary during early development (15–38 months) and their genetic relationship with mid-childhood reading and cognitive skills (7–8 years), based on longitudinal phenotype measures and genome-wide genetic data from up to 6,524 unrelated children. We showed that early-life vocabulary skills are influenced by multiple independent genetic factors, of which two also relate to mid-childhood skills, suggesting developmental stability. One genetic factor emerging with expressive vocabulary at 24 months was linked to subsequent verbal abilities, including vocabulary measures at 38 months, as well as mid-childhood reading and verbal intelligence performance. A second, independent genetic factor related to receptive vocabulary at 38 months contributed more generally to variation in mid-childhood reading, verbal and non-verbal intelligence. Thus, the genetic foundations of mid-childhood reading and cognitive abilities involve at least two independent genetic factors that emerge during early-life language development and may implicate differences in overarching cognitive mechanisms.

## Introduction

The number of words produced and understood by children during the first few years of life is a rapidly changing developmental phenotype that is often used to assess the level of language acquisition [1]. One of the first precursors of expressive vocabulary (i.e. word production) in typically developing children is canonical babbling, which emerges around the age of four to six months [2], followed by the spontaneous production of first words between 10 to 15 months of age [3]. With progressing development, the number of produced words increases, reaching a median of 40 words at 16 months [1], often trailed by a period of rapid growth till the age of about 22 months [4] and a steady increase after that. This results in the production of approximately 500 words at 30 months [5] and about 2,600 words at six years of age [6]. The development of receptive vocabulary (i.e. word comprehension) typically precedes expressive vocabulary in developing children [7], with the understanding of the first few words emerging between 6 to 9 months of age [8]. Thus, receptive vocabulary is usually larger than expressive vocabulary in size [7]. For example, the number of words understood by infants at 16 months of age has a median of 169 words, and is, thus, approximately 129 words larger compared to their expressive vocabulary at the same time [1]. This discrepancy increases during development, with a receptive vocabulary size of about 20,000 to 24,000 words at the age of six years, which is about six times larger than its expressive counterpart [6].

The rate of language acquisition, including vocabulary size, varies between children during early language development [9,10]. These large interindividual differences can partially be explained by genetic variation. Twin studies have estimated that genetic influences account for 17% to 25% of variation in expressive vocabulary at 24 months [11,12], 10% to 14% of variation in expressive vocabulary at 36 months [11] and 28% of variation in receptive vocabulary at 14 months [13]. Studies using genotype data from unrelated children provided similar estimates, with single-nucleotide polymorphism heritability (SNP-h^2^) estimates of 13% to 14% for expressive vocabulary at 15 to 30 months of age [14] and 12% for receptive vocabulary at 38 months of age [15].

Despite some stable genetic contributions during early development, there is also evidence for age-specific genetic influences on vocabulary skills. For example, measures of expressive vocabulary size assessed between 15 and 36 months are genetically moderately correlated based on samples of unrelated individuals as well as twins, with estimates ranging from 0.48 to 0.69 [11,14]. Additionally, a considerable proportion (3% to 28%) of the total variation in early expressive language assessed at 24, 36 and 48 months could be explained by measure-specific additive genetic variance and not by a shared latent factor [16]. However, the field is still missing a comprehensive characterisation of the genetic architecture underlying early-life vocabulary development that characterises age-specific genetic influences across infancy and toddlerhood starting from the first-word stage as well as differences between receptive and expressive language skills.

Genetic links between early language processes (assessed from 24 to 48 months of age) and subsequent language- and literacy-related abilities (assessed from mid-childhood to adolescence) have been reported by studies of both twins and unrelated individuals [15–17]. This research suggested that genetic variance in mid-childhood/adolescent language, literacy and cognitive development can already be captured by genetic factors contributing to language skills in toddlerhood, i.e. before the age of four years. More specifically, genetic influences underlying receptive vocabulary at 38 months could capture, through amplification, the majority of genetic variation contributing to a wide spectrum of mid-childhood/early-adolescent literacy, verbal and non-verbal cognitive skills in a sample of unrelated individuals [15]. So far, however, our understanding of the developmental origin of these factors is incomplete.

Here, we (i) examine stability and change in the developmental genetic architecture of language during the first three years of life and (ii) assess evidence for emerging genetic associations with mid-childhood verbal and non-verbal abilities during different stages of early-life vocabulary development. We model multivariate genetic architectures underlying these traits as directly captured by genome-wide information (based on genetic-relationship-matrices, GRMs) for up to 6,524 unrelated youth from the UK Avon Longitudinal Study of Parents and Children (ALSPAC) birth cohort [18,19]. We apply Genetic-relationship-matrix structural equation modelling (GSEM) [20], analogous to twin research-modelling techniques, and dissect the phenotypic variation into additive genetic and residual variance structures.

## Results

### Analysis strategy

A two-stage analysis strategy was followed: During the first stage of the analysis (Stage 1), we examine the multivariate genetic variance structure of expressive and receptive vocabulary from 15 to 38 months of age (Table 1). A structural equation model (SEM) following a Cholesky decomposition was fitted to all vocabulary measures with at least nominal evidence for SNP-h^2^ (*P*<0.05). During the second stage (Stage 2), we extended these Cholesky decomposition models, and assessed the emerging genetic links between early-life vocabulary (15 to 38 months) and reading, verbal intelligence quotient (VIQ) scores and performance (non-verbal) intelligence scores (PIQ) during mid-childhood (7 to 8 years of age, S1 Table). Applying a Cholesky decomposition approach enabled us to study longitudinal relationships between measures in a hypothesis-free manner, while allowing for both shared (i.e. across age and/or ability) and unique (i.e. age- and ability-specific) genetic influences. Specifically, we model genetic factor structures underlying bivariate genetic correlation patterns. For all SEMs studied, we report path coefficients (the square root of individual factor variance contributions) and the corresponding percentage of explained phenotypic variance, in addition to total SNP-h^2^, genetic and residual correlations, factorial co-heritability (the proportion of total SNP-h^2^ explained by a specific genetic factor) and bivariate heritability (the contribution of genetic factors to the observed phenotypic correlation between two measures) (S3, S4, S5 Text).

### Stage 1: The developmental genetic architecture of early-life vocabulary skills

#### Univariate SNP-heritability estimates for early-life vocabulary measures

Measures of early-life language included expressive vocabulary at 15, 24 and 38 months and receptive vocabulary at 15 and 38 months (Table 1). They were assessed with parent-reported questionnaires and analysed as rank-transformed scores (see Methods, S2 Table). For comparison with multivariate models, we first estimated SNP-h^2^ using Genome-based Restricted Maximum Likelihood as implemented in Genome-wide Complex Trait Analysis (GCTA) software [21]. Common genetic variation accounted for a modest proportion of phenotypic variation in early-life vocabulary throughout, except for receptive vocabulary at 15 months, where SNP-h^2^ was consistent with zero (Table 1). GCTA-SNP-h^2^ estimates for expressive vocabulary at 15, 24 and 38 months were 11%(SE = 5%), 16%(SE = 6%) and 18%(SE = 6%), respectively. For receptive vocabulary at 15 and 38 months, SNP-h^2^ was estimated at 8%(SE = 5%) and 12%(SE = 6%), respectively. Given little evidence for SNP-h^2^ for receptive vocabulary at 15 months (*P*>0.05; Table 1), we excluded this measure from further correlation and GSEM analyses to facilitate the convergence of the models. Note that it was not possible to include the receptive vocabulary score at 24 months due to discrepancies in the questionnaire coding scheme (see Methods).

**Table 1:** Early-life expressive and receptive vocabulary in ALSPAC.

Measure	Psychological instrument	Mean score (SE)	Mean age (SE) in months	N (%male)	GCTA-SNP-h^2^ (SE)
Expressive vocabulary	MacArthur CDI^a^	14.29 (17.76)	15.41 (0.97)	6,524 (51.1)	0.11 (0.05)
	MacArthur CDI^b^	64.21 (35.11)	24.39 (1.03)	6,014 (51.7)	0.16 (0.06)
	MacArthur CDI^b^	113.33 (17.44)	38.48 (1.17)	6,092 (51.4)	0.18 (0.06)
Receptive vocabulary	MacArthur CDI^a^	75.85(31.78)	15.41 (0.97)	6,524 (51.1)	0.08 (0.05)
	MacArthur CDI^b^	109.75 (23.75)	38.48 (1.17)	6,092 (51.4)	0.12 (0.06)

Expressive vocabulary and receptive vocabulary were assessed between 15–38 months of age in independent children (genetic relationship<0.05).

^a.^Adapted form of the MacArthur CDI:Words & Gestures, consisting of 134 words.

^b.^Adapted from of the MacArthur CDI:Words & Sentences, consisting of 123 words.

Abbreviations: ALSPAC, Avon Longitudinal Study of Parents and Children; CDI, Communicative Development Inventory; GCTA, Genome-wide Complex Trait Analysis; h^2^, heritability; SNP, single-nucleotide polymorphism.

#### Bivariate phenotypic and genetic correlations among early-life vocabulary measures

**Fig 1:**
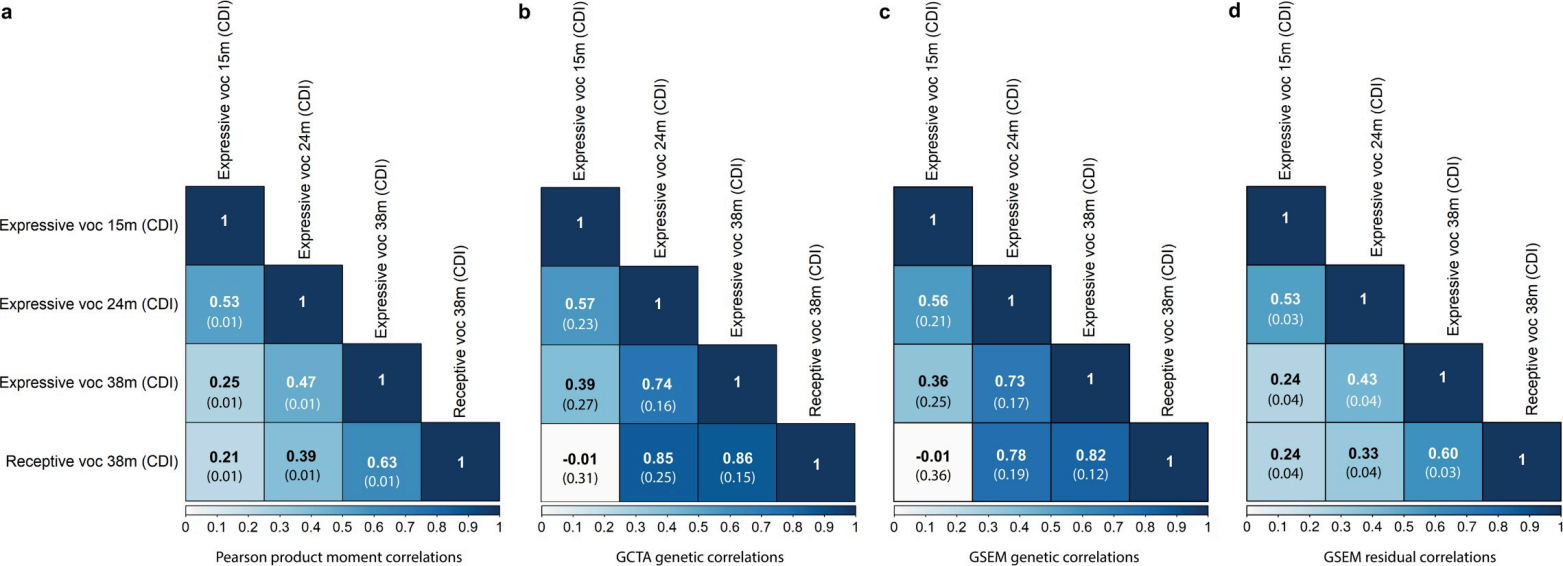
Phenotypic, genetic and residual correlations among early-life vocabulary scores (15 to 38 months). Correlation patterns are shown for rank-transformed measures with sufficient evidence for SNP-h^2^ (P<0.05) Standard errors are shown in brackets. **(A)** Phenotypic correlations were estimated with Pearson correlation coefficients. **(B)** GCTA genetic correlations based on GREML. **(C)** GSEM genetic correlations. **(D)** GSEM residual correlations. Abbreviations: CDI, Communicative Development Inventory; GCTA, Genome-based Restricted Maximum Likelihood as implemented in genome-wide complex trait analysis (GCTA) software; GREML, Genome-based restricted maximum likelihood; GSEM, genetic-relationship-matrix structural equation modelling; m, months; voc, vocabulary. Genetic analyses were conducted using genetic relationship matrices based on directly genotyped SNPs and individuals with a genetic relationship of <0.05.

Early-life vocabulary measures were phenotypically interrelated, although correlations decreased with increasing age windows (Fig 1A). The largest phenotypic correlation (r_p_) was estimated between expressive and receptive vocabulary at 38 months (r_p_ = 0.63). Bivariate genetic correlations (r_g_) among early-life vocabulary measures emerged from 24 months of age onwards (Fig 1B). Mirroring phenotypic relationships, the largest genetic correlation was observed between expressive and receptive vocabulary assessed at 38 months (GCTA-r_g_ = 0.86(SE = 0.15), *P* = 0.004).

#### Multivariate genetic variance structures between early-life vocabulary measures

Using GSEM, we studied the multivariate genetic architecture underlying early vocabulary development. A multivariate SEM was fitted to expressive vocabulary at 15, 24 and 38 months as well as receptive vocabulary at 38 months (in this order), following a Cholesky decomposition. SNP-h^2^ estimates were nearly identical for all early-life vocabulary measures using univariate GCTA and multivariate GSEM approaches (S2 Table). Estimated bivariate genetic correlations using GSEM were also highly consistent with GCTA findings (Fig 1B and 1C), with overlapping 95%-confidence intervals (95%-CIs). GSEM-estimated residual correlations among vocabulary measures were modest to moderate (Fig 1D), suggesting further shared aetiological mechanisms not captured by common variation.

**Fig 2:**
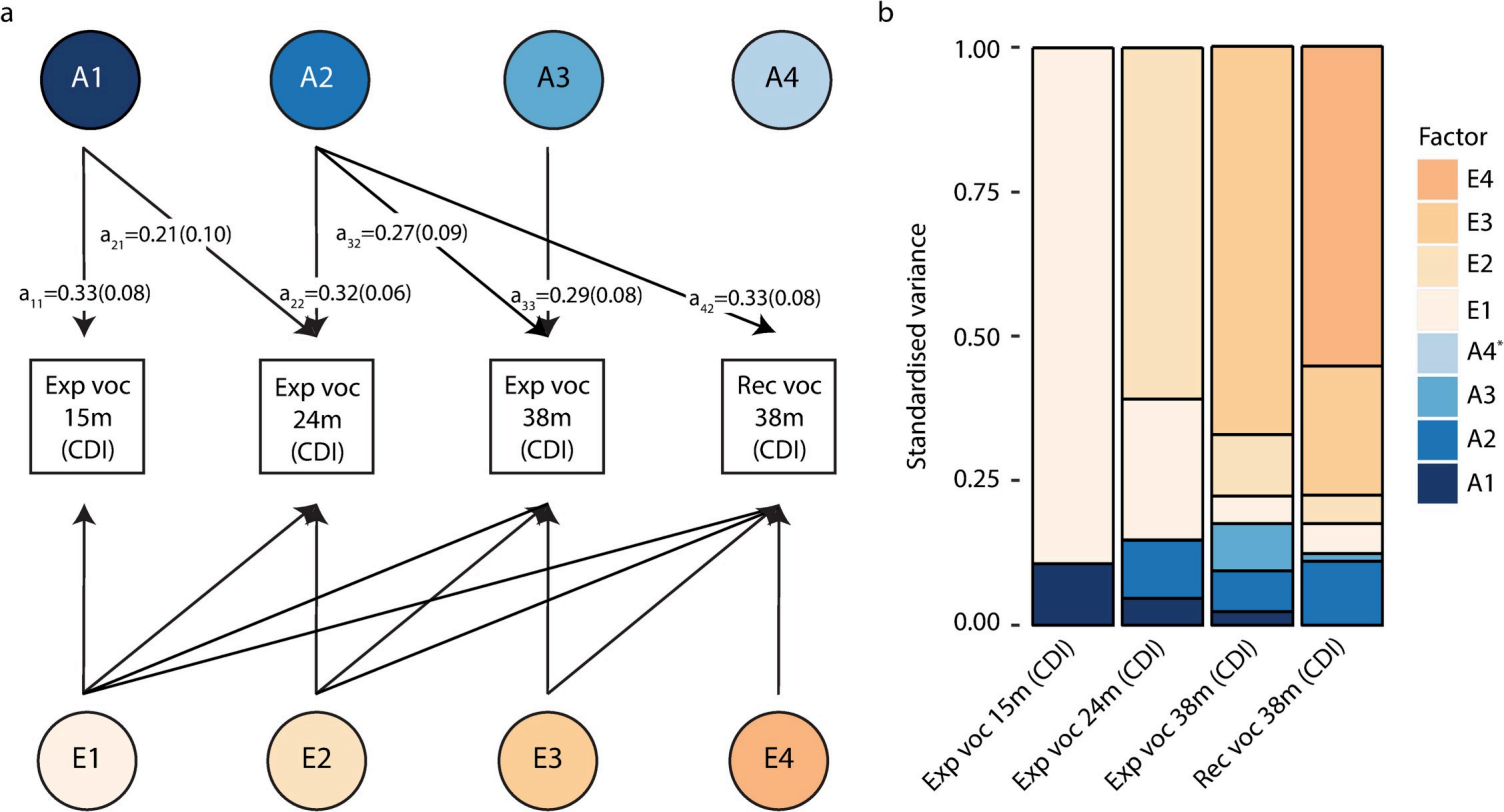
Structural model of early-life vocabulary scores (15 to 38 months). Genetic-relationship matrix structural equation modelling (GSEM) of rank-transformed early-life vocabulary scores (15, 24 and 38 months of age) based on all available observations for children across development (N≤6,524; Cholesky decomposition model). **(A)** Path diagram with standardised path coefficients and corresponding standard errors. Only paths with a path coefficient passing a P-value threshold of 0.05 are shown. Full information on path coefficients and their standard errors can be found in S3 Table. **(B)** Standardised variance explained by genetic and residual factors modelled in (A). * The proportion of phenotypic variance explained by genetic factor A4 in receptive vocabulary at 38 months is negligible. Abbreviations: CDI, Communicative Development Inventory; Exp, expressive; m, months of age; Rec, receptive; voc, vocabulary.

Structural models of vocabulary measures assessed during the first three years of life revealed that the underlying genetic architecture is dynamic, with evidence for age-specific genetic influences in addition to genetic influences that were stable during early development (Fig 2). The first genetic factor (A1) accounted for 10.6%(SE = 5.0%) of the phenotypic variation in expressive vocabulary at 15 months (Fig 2, S4 Table), which can be approximated by squaring the corresponding estimated standardised path coefficient, here a_11_ (path coefficient a_11_:0.33(SE = 0.08), *P* = 2x10^-5^). By structural model design, the phenotypic variance explained by a_11_ corresponds to the SNP-h^2^ of expressive vocabulary at 15 months (S3 and S4 Tables). Genetic factor A1 was also related to expressive vocabulary at 24 months (path coefficient a_21_:0.21(SE = 0.10), *P* = 0.04), explaining 4.6%(SE = 4.4%) of the phenotypic variation and accounting for almost a third of the SNP-h^2^ (factorial co-heritability: 31.2%(SE = 23.4%), S4 Table). However, there was little evidence for shared genetic influences between expressive vocabulary at 15 months and either expressive or receptive vocabulary scores at 38 months (Fig 2, S4 Table). This pattern of findings suggests that genetic influences underlying expressive vocabulary at 15 months play a decreasing role during the course of later vocabulary development, consistent with data from genetic correlation and bivariate heritability analyses (Fig 1B and 1C, S6 Table).

Expressive vocabulary at 24 months also loaded on a second genetic factor (A2), explaining an additional 10.1%(SE = 4.0%) of the phenotypic variation (path-coefficient a_22_: 0.32(SE = 0.06), *P* = 4x10^-7^; Fig 2, S4 Table) and the majority of the SNP-h^2^ (factorial co-heritability: 68.8%(SE = 23.4%), S5 Table). This genetic factor was also shared with both expressive (path coefficient a_32_:0.27(SE = 0.09), *P* = 0.005) and receptive (path coefficient a_42_:0.33(SE = 0.08), *P* = 4x10^-5^) vocabulary at 38 months, accounting for 7.1%(SE = 5.0%) and 11.0%(5.3%) of the phenotypic variation, respectively (Fig 2, S4 Table). For receptive vocabulary at 38 months, this genetic factor captured the majority of the SNP-h^2^ (factorial co-heritability: 88.9%(SE = 23.1%), S4 Table), suggesting a largely shared genetic aetiology with expressive vocabulary at 24 months, as confirmed by their high genetic correlation (GSEM-r_g_ = 0.78(SE = 0.19), Fig 1C).

The third genetic factor (A3) was related only to expressive vocabulary at 38 months (path coefficient a_33_:0.29(SE = 0.08), *P* = 0.001) and explained 8.2%(SE = 4.9%) of the phenotypic variation (Fig 2, S4 Table), corresponding to nearly half of the SNP-h^2^ (factorial co-heritability: 47.0%(SE = 25.1%), S5 Table). This genetic factor was unrelated to receptive vocabulary at 38 months (path coefficient a_43_:0.12(SE = 0.12), *P* = 0.35). Thus, it is likely that the genetic correlation between expressive and receptive vocabulary at 38 months (GSEM-r_g_ = 0.82(SE = 0.12, Fig 1C) is primarily driven by genetic variance shared with expressive vocabulary at 24 months.

Finally, there was little support for the presence of a fourth genetic factor (A4) that would be exclusively related to receptive vocabulary at 38 months (Fig 2, S4 Table). However, according to findings from our previous work, such a factor is likely to account only for very little phenotypic variance in receptive vocabulary at 38 months [15]. Therefore, it may only become detectable once modelled together with other heritable traits sharing these genetic influences.

### Stage 2: Multivariate genetic variance structures between early-life vocabulary and mid-childhood reading, verbal and performance intelligence

In a second step, we assessed the emergence of genetic links with mid-childhood reading accuracy/comprehension at 7 years, verbal intelligence quotient scores (VIQ) at 8 years and performance intelligence quotient scores (PIQ) at 8 years (S1 Table) across the studied vocabulary measures during the first three years of life, using rank-transformed measures. The selected measures of reading and verbal intelligence are representative of previously reported genetic association patterns between vocabulary at 38 months and a wide spectrum of language, literacy and cognitive abilities in ALSPAC [15]. We contrast these verbal abilities with a measure of non-verbal intelligence (PIQ) to evaluate differences in developmental association patterns of these different components of general cognitive ability with respect to early-life vocabulary. Thus, the model from the first step (Fig 2) was extended to include, in turn, each of the three mid-childhood skills, resulting in three further SEMs (with measures included in chronological order).

**Fig 3:**
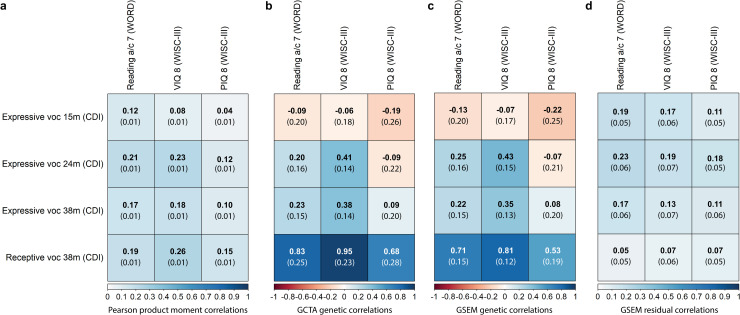
Phenotypic, genetic and residual correlations between early-life vocabulary scores and mid-childhood reading, verbal intelligence and performance intelligence. Correlation patterns are shown for rank-transformed measures with sufficient evidence for SNP-h^2^ (*P*<0.05) Standard errors are shown in brackets. **(A)** Phenotypic correlations were estimated with Pearson correlation coefficients. **(B)** GCTA genetic correlations based on GREML. **(C)** GSEM genetic correlations. **(D)** GSEM residual correlations. Abbreviations: a, accuracy; c, comprehension; CDI, Communicative Development Inventory; GCTA, Genome-based Restricted Maximum Likelihood as implemented in genome-wide complex trait analysis (GCTA) software; GREML, Genome-based restricted maximum likelihood; GSEM, genetic-relationship-matrix structural equation modelling; m, months; PIQ; performance intelligence quotient; VIQ; verbal intelligence quotient, voc, vocabulary; WISC-III, Wechsler Intelligence Scale for Children III; WORD, Wechsler Objective Reading Dimension. Genetic analyses were conducted using genetic relationship matrices based on directly genotyped SNPs and individuals with a genetic relationship of <0.05.

At the phenotypic level, all early-life vocabulary measures showed low to modest correlations with both mid-childhood verbal and non-verbal skills (Fig 3A), with the largest phenotypic correlation between receptive vocabulary at 38 months and VIQ at 8 years (r_p_ = 0.26). The selected mid-childhood skills, reading, VIQ and PIQ, were all moderately heritable, with GCTA-SNP-h^2^ estimates of 42%(SE = 6%), 54%(SE = 7%) and 26%(SE = 7%), respectively. These estimates largely corresponded to GSEM-SNP-h^2^ estimates (S3 Table). Using GCTA, bivariate genetic correlations of mid-childhood skills with early-life vocabulary measures (Fig 3B) revealed moderate genetic correlations of VIQ with expressive vocabulary at 24 (GCTA-r_g_ = 0.41(SE = 0.14),*P* = 0.003) and 38 months (GCTA-r_g_ = 0.38(SE = 0.14),*P* = 0.003), but high genetic correlations of both verbal and non-verbal skills with receptive vocabulary at 38 months (reading GCTA-r_g_ = 0.83(SE = 0.25), *P* = 9x10^-6^; VIQ GCTA-r_g_ = 0.95(SE = 0.23), *P* = 1x10^-8^; PIQ GCTA-r_g_ = 0.68(SE = 0.28), *P* = 0.004). GCTA and GSEM genetic correlation estimates were highly consistent, with overlapping 95%-CIs (Fig 3B and 3C). GSEM-estimated residual correlations between early-life and mid-childhood measures were low (Fig 3D).

**Fig 4:**
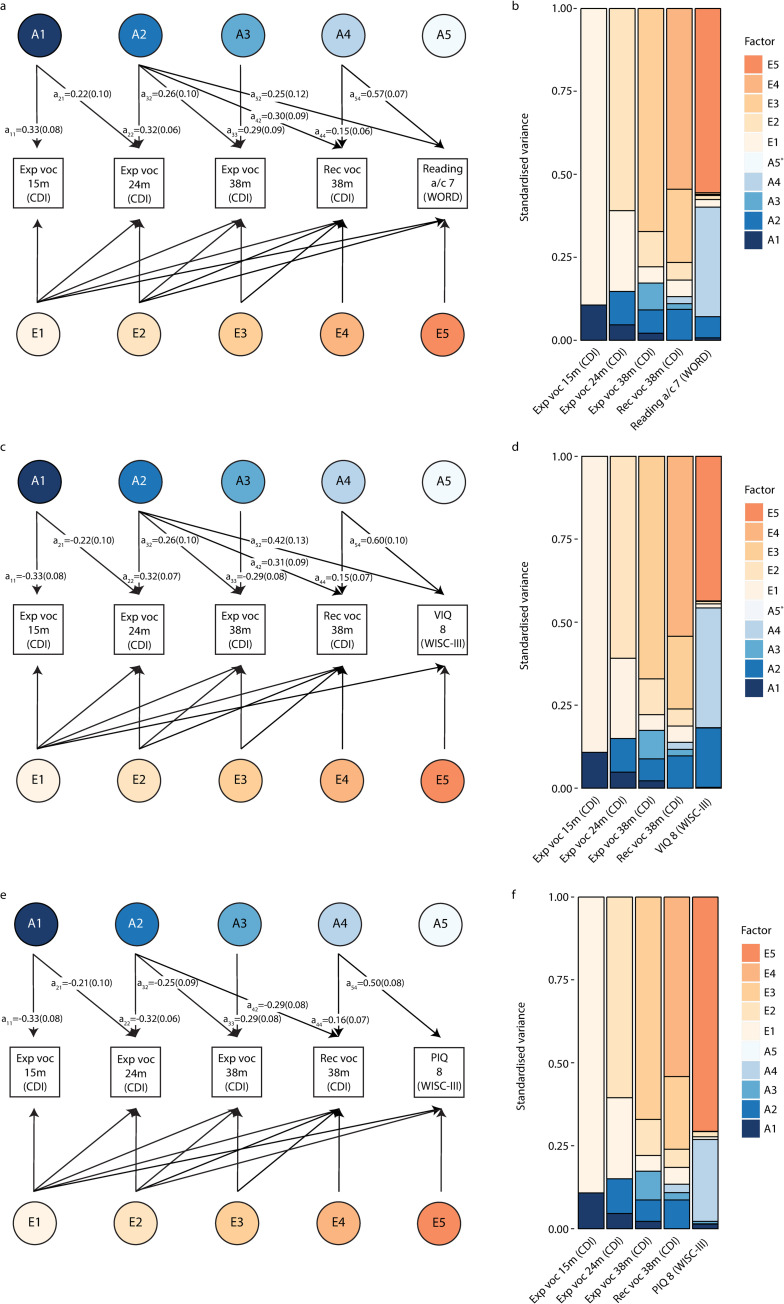
Structural models of early-life vocabulary and mid-childhood reading and cognition. Genetic-relationship matrix structural equation modelling (GSEM) of rank-transformed early-life vocabulary scores (15, 24 and 38 months of age) in combination with rank-transformed mid-childhood scores capturing (**A,B**) reading accuracy/comprehension at 7 years, (**C,D**) VIQ at 8 years or (**E,F**) PIQ at 8 years, based on all available observations for children across development (N≤6,524). **(A,C,E)** Path diagrams with standardised path coefficients and corresponding standard errors including mid-childhood **(A)** reading accuracy/comprehension, **(C)** VIQ and **(E)** PIQ outcomes. Only paths with a path coefficient passing a *P*-value threshold of 0.05 are shown. Full information on all path coefficients and their standard errors can be found in S7, S8, S9 Tables. **(B,D,F)** Standardised variance explained by genetic and residual factors as modelled in a,c,e for models including **(B)** reading accuracy/comprehension, **(D)** VIQ, and **(F)** PIQ. * The proportion of phenotypic variance explained by genetic factor A5 is negligible. Abbreviations: a, accuracy; c, comprehension; CDI, Communicative Development Inventory; Exp, expressive; m, months of age; Rec, receptive; PIQ, performance intelligence quotient; VIQ, verbal intelligence quotient; voc, vocabulary; WISC-III, Wechsler Intelligence Scale for Children III; WORD, Wechsler Objective Reading Dimension.

Using multivariate structural models, our results showed, first, that there is little evidence for genetic links between expressive vocabulary at 15 months (A1) and vocabulary, reading or cognition abilities after the age of 24 months (Fig 4, S7, S8 and S9 Tables). Second, the developmentally novel genetic factor emerging for expressive vocabulary at 24 months (A2), explained further genetic variance in receptive and expressive vocabulary at 38 months (as outlined above) and, importantly, mid-childhood verbal skills. Specifically, it was related to both reading accuracy/comprehension (path coefficient a_52_:0.25(SE = 0.12), *P* = 0.04) and VIQ (path coefficient a_52_ = 0.42(SE = 0.13), *P* = 0.001), and accounted for 6.4%(6.2%) and 17.9%(11.1%) of their phenotypic variation, respectively (Fig 4, S7 and S8 Tables). Consequently, it is likely that this genetic factor contributes to the genetic correlation of VIQ with vocabulary measures at 24 and 38 months, as well as the genetic correlation of reading accuracy/comprehension at 7 years with receptive vocabulary at 38 months (Fig 3). Genetic factor A2 was not linked to PIQ at 8 years (path coefficient a_52_:-0.03(SE = 0.12), *P* = 0.78)(Fig 4E and 4F, S9 Table). These findings may reflect some genetic specificity for verbal skills (reading and VIQ), compared to non-verbal cognition, though the 95%-CIs for the identified path coefficients overlap (path coefficients a_52_-reading accuracy/comprehension: 95%-CI = 0.01–0.49, a_52_-VIQ: 95%-CI = 0.17–0.68, a_52_-PIQ: 95%-CI = -0.26–0.20, derived assuming normality). Third, genetic influences identified for expressive vocabulary at 38 months (A3) were unrelated to receptive vocabulary assessed at the same age (as outlined above) and later mid-childhood abilities (Fig 4, S7, S8, S9 Tables). Thus, the genetic correlation between expressive vocabulary at 38 months and mid-childhood VIQ (GSEM-r_g_ = 0.35(SE = 0.13), Fig 3C) is primarily driven by genetic variance shared with expressive vocabulary at 24 months. Fourth, joint modelling of early-life vocabulary measures with mid-childhood abilities enabled the identification of a genetic factor that affects receptive vocabulary at 38 months (A4) and that is independent of early-life expressive vocabulary genetic factors (path coefficient a_44_:0.15(SE = 0.07), *P* = 0.04, Fig 4C). Although this genetic factor accounted for only a tiny proportion of the phenotypic variation in receptive vocabulary at 38 months (2.1%(SE = 1.9%)), it explained 33.0%(SE = 8.2%), 36.1%(SE = 11.5%) and 24.7%(SE = 7.5%) of the phenotypic variation in reading accuracy/comprehension, VIQ and PIQ, respectively (path coefficients a_54_-reading accuracy/comprehension:0.57(SE = 0.07), *P*<1x10^-10^; a_54_-VIQ: 0.60(0.10), *P* = 3x10^-10^; a_54_-PIQ: 0.50(0.08), *P*<1x10^-10^). The genetic variance explained by genetic factor A4 corresponds to the majority of the estimated SNP-h^2^ for mid-childhood abilities, as indicated by factorial co-heritabilities (reading: 82.3%(SE = 16.1%), VIQ: 66.4%(SE = 19.9%), PIQ: 91.8%(SE = 15.1%), S10 Table). Finally, there was little evidence for novel genetic factors emerging during mid-childhood (A5, Fig 4), consistent with previous findings [15]. Thus, the fitted multivariate models for early-life vocabulary and mid-childhood skills were consistent with both the identified multivariate genetic architecture of early-life vocabulary (Fig 2) and the previously reported amplification of genetic factors for vocabulary at 38 months [15].

The phenotypic covariance of mid-childhood reading, VIQ and PIQ with receptive vocabulary at 38 months (Fig 3A) was primarily due to genetic covariance, with bivariate heritability estimates of 0.87(SE = 0.21), 0.88(SE = 0.16) and 0.68(SE = 0.27), respectively (S11 Table). This is consistent with little evidence for residual correlation between receptive vocabulary at 38 months and mid-childhood measures (Fig 3D). For verbal mid-childhood skills, such as VIQ, evidence for bivariate heritability with early-life expressive vocabulary was already detectable at 24 months of age (bivariate heritability: 0.54(SE = 0.19)), as well as at 38 months of age (bivariate heritability: 0.60(SE = 0.24)).

## Discussion

This genome-wide longitudinal analysis of vocabulary size during the first three years of life assessed in unrelated children demonstrates that the genetic architecture underlying expressive and receptive vocabulary is dynamic, with evidence for both age- and ability-specific genetic influences in addition to genetic influences that were stable during early development. Genetic continuity into mid-childhood was found for two independent early-life genetic factors, which contribute to the genetic variance of reading and cognitive skills at 7 and 8 years of age, respectively. One stable early-life genetic source of variation was related to expressive vocabulary and emerged at 24 months of age, accounting for up to 11% of the phenotypic variation in vocabulary measures at 38 months, as well as between 6.4% and 17.9% of the phenotypic variation in mid-childhood abilities, especially verbal skills such as reading and VIQ. A second, independent and stable early-life genetic factor was identified for receptive vocabulary at 38 months and explained between 24.7% and 36.1% of the phenotypic variance in both mid-childhood verbal and non-verbal cognitive abilities, including PIQ, corresponding to the majority of SNP-h^2^ (≥66%). Given the modest SNP-h^2^ of early-life vocabulary scores, ranging from 11% to 18%, this suggests not only genetic stability, but also an amplification of early genetic variance during the life-course that contributes to the markedly increased SNP-h^2^ of later-life reading and cognition (27% to 54%).

The identification of multiple independent genetic factors related to vocabulary during the first three years of life may reflect rapid changes in mastering behavioral and language skills. Genetic influences identified for expressive vocabulary at 15 months (A1) were also related to expressive vocabulary at 24 months, but were not linked to vocabulary, reading or cognition measures beyond this age. Thus, these early genetic influences might primarily affect the very first stages of language development that, once achieved, have little impact on subsequent verbal and cognitive development. A plausible candidate process for this is the acquisition of phonological skills to identify phonemes and sequences from speech and their storage for future production. Almost all typically developing children have mastered these skills at the age of 24 months, resulting in little contribution to the variation observed in expressive vocabulary from this age onwards [22].

The stable independent genetic factor emerging for expressive vocabulary at 24 months (A2) contributes to the genetic architectures underlying verbal processes throughout childhood, in contrast to genetic factor A1. Specifically, the genetic influences captured by A2 were related to both expressive and receptive vocabulary at 38 months, as well as mid-childhood verbal abilities such as reading and VIQ, but not PIQ (although 95%-CIs of estimated genetic path coefficients overlap with those for PIQ). This genetic factor may reflect stages of language learning that take place after the production of words in isolation at the age of 10 to 15 months [3]. This includes, for example, an increasing vocabulary size as well as the use of more complex grammatical structures, marked by the emergence of two-word combinations around the age of 18 to 24 months [1,23]. It has been shown that lexical and grammatical development share underlying acquisition mechanisms [24] and measures of expressive vocabulary and grammatical development at two and three years of age are both phenotypically and genetically correlated [11].

Expressive vocabulary at 38 months loaded on an additional independent genetic factor (A3) that was not related to receptive vocabulary at the same age, nor to any of the studied mid-childhood reading and IQ measures. This genetic factor may, thus, involve genetic associations with processes that affect expressive vocabulary at an early age, but do not play a role in later cognition. They may, for example, reflect social abilities, which are known to impact on vocabulary development and vice versa [25]. Note that expressive vocabulary at 38 months is nonetheless genetically related to mid-childhood verbal processes due to shared genetic influences that were already detectable at 24 months (A2).

The majority of SNP-h^2^ for mid-childhood reading, VIQ and also PIQ was accounted for by a genetic factor that emerged at 38 months of age for receptive vocabulary (A4), consistent with previous findings [15]. Although this stable genetic factor explained only a very small part of the phenotypic variance in receptive vocabulary (2.1%), it accounted from 66% to 92% of the phenotypic variation in later reading performance, verbal and non-verbal cognition, with very little residual contributions. Due to the wide spectrum of associated mid-childhood phenotypes that are linked with this genetic factor, including both later verbal and non-verbal cognitive abilities, it is possible that the genetically encoded biological processes are important for cognitive development in general. It merits noting that the genetic factor A4 was only detectable once modelled together with a mid-childhood skill sharing underlying genetic variance, probably due to the low proportion of phenotypic variation that it explained in early-life receptive vocabulary.

Previous twin studies demonstrating genetic links between language use in early childhood and later language/literacy skills have been based on a latent factor approach jointly capturing genetic variance of expressive language skills between the ages of 2 and 4 years [16,17]. Here, we used a sample of unrelated children with genome-wide genotyping data and distinguish language measures during the first three years of life based on both modality and age at assessment. We extend and refine the previous twin findings by showing that (i) early-life expressive vocabulary at 15 months of age is influenced by a genetic factor that is only shared across expressive vocabulary scores during infancy, and (ii) that there are at least two independent genetic factors during early life that are associated with mid-childhood reading and cognition. Genetic associations with mid-childhood verbal cognitive processes arise as early as 24 months of age, whereas genetic influences that are relevant for mid-childhood general cognitive development emerge as early as 38 months of age for receptive vocabulary, and are independent of expressive vocabulary. This latter distinction is important as receptive vocabulary at 38 months also shares a genetic factor with expressive vocabulary at 24 months and subsequent reading and verbal intelligence. The diversity in genetic factors may implicate differences in overarching cognitive processes that are already detectable during toddlerhood. This is important as genetic influences associated with early-life vocabulary could fully account for the SNP-h^2^ of mid-childhood reading, verbal and non-verbal intelligence. The presence of such genetic stability implicating verbal processes and general cognition from toddlerhood to, at least, mid-childhood may, furthermore, suggests shared biological underpinnings. Thus, joint genome-wide association study analyses across developmental stages may facilitate an increase in study power.

In addition to the strengths of this study described above, this study benefits from modelling multivariate genetic variance structures in unrelated individuals directly, based on genome-wide information, using novel structural equation modelling techniques. It is, however, not possible to infer biological mechanisms underlying the identified genetic factor structures with the current methodology. We furthermore exploit the phenotypic richness of the ALSPAC cohort, including longitudinally assessed vocabulary measures during early development as well as reading and cognitive outcomes in mid-childhood. However, we did not study genetic factors underlying receptive vocabulary assessed at 15 months in a multivariate context due to little evidence for SNP-h^2^ (SNP-h^2^: 0.08(SE = 0.05), *P*>0.05). In general, parental judgement of children’s early language comprehension is thought to be less reliable than parental judgement of children’s language production [1,26]. Assessing receptive language skills at this stage requires that parents notice their children’s subtle and often idiosyncratic non-verbal responses to words and phrases, and it is, therefore, less objectively defined. This might result in a relatively high random error rate for receptive vocabulary scores at 15 months, reducing power [27]. This study has also several limitations. First, given the rapidly changing nature of early vocabulary size, increasingly larger and complex word lists are required to reliably assess vocabulary size at 24 and 38 months compared to 15 months of age. Thus, the observed differences in genetic factor structures during early life may reflect differences in CDI instruments, although this is unlikely to fully explain our findings, given substantial phenotypic correlations between expressive vocabulary scores at 15 and 24 months of age (r_p_ = 0.53). To jointly analyse measures with different score distributions, including positive and negative skews, all measures were rank-transformed. Thus, the presented SEMs reflect multivariate variance structures of rank-transformed scores. In addition, vocabulary assessments at 38 months of age might be affected by ceiling effects (S2 Table), as the MacArthur CDI:Words & Sentences was developed for children up to 30 months [5]. This may have reduced phenotypic variation and, thus, power to detect genetic variance components at 38 months. Second, it has recently been shown that heritability and genetic relationships estimated in samples of unrelated individuals, especially for cognition-related traits [28,29], might be inflated by indirect genetic effects, reflecting a type of gene-environment correlation [30]. The observed association patterns between early-life vocabulary and mid-childhood reading and cognitive skills may therefore represent both shared genetic variance and indirect genetic effects. Future research using family-based data is warranted to assess the impact of indirect genetic effects on the reported association patterns. Third, the sparsity of large data sets with longitudinal information on expressive and receptive vocabulary during infancy and toddlerhood, in addition to genome-wide data, currently prevents a direct replication of our findings in independent cohorts.

Taken together, our findings reveal a dynamic genetic landscape underlying vocabulary during the first three years of life, including a developmentally stable genetic factor related to vocabulary measures at 24 and 38 months of age. Furthermore, we found evidence for genetic continuity of two independent early-life genetic factors that contribute to both verbal and general cognitive abilities in mid-childhood. Thus, the genetic foundations for both mid-childhood reading and cognition lie in toddlerhood, but are diverse, and may implicate aetiological differences in overarching cognitive processes that are detectable long before the age of schooling.

## Methods

### Sample description and trait selection

#### Ethics Statement

Ethical approval was provided by the ALSPAC Ethics and Law Committee and the Local Research Ethics Committees. Written informed consent was obtained where appropriate for questionnaire and clinical data from participants following recommendations of the ALSPAC Ethics and Law Committee at the time. Consent for biological samples was collected in accordance with the Human Tissue Act (2004).

#### Cohort information

Participants were born in 1991 or 1992 and included in ALSPAC, a UK population-based birth cohort (S1 Text)[18,19].

#### Genetic analyses

Genotyping and genotype calling was performed using the Illumina HumanHap550 quad chip and Illumina GenomeStudio software. Quality control of genetic data was applied using PLINK (v1.07)[31] at both the SNP and individual level following standard procedures. Individuals were excluded in case of gender mismatch between reported and genetic sex information, >3% missing SNP information, non-European ancestry, or interindividual relatedness (genomic relatedness>0.05). SNPs were excluded if they had a low call rate (<99%), were rare (<1%) and/or deviated from Hardy-Weinberg equilibrium (*P*<5x10^-7^). After quality control, 7,924 children and 465,740 SNPs with high-quality genetic data remained.

#### Early-life vocabulary measures

Expressive and receptive vocabulary was assessed at 15, 24 and 38 months of age using parental-reports (predominantly mother) of age-specific defined word lists adapted from the MacArthur Communicative Development Inventory (CDI). At 15 months of age, expressive and receptive vocabulary were assessed with an abbreviated version of the MacArthur CDI:Words & Gestures (133 words, 8 to 16 months of age)[32]. Expressive vocabulary scores were recorded as the number of words a child could “say and understand”, while receptive vocabulary scores were recorded as the number of words a child could either “understand” only or “say and understand”. At 24 and 38 months of age, an abbreviated vocabulary list from the MacArthur CDI:Words & Sentences (123 words, 16–30 months of age)[5] was used. At both ages, expressive vocabulary was measured as the total number of words a child could either “say” only or “say and understand”. Receptive vocabulary at 38 months was measured as the total number of words a child could either “understand” only or “say and understand”. The receptive vocabulary score at 24 months was excluded due to discrepancies in the applied coding scheme (excluding words a child could “say and understand”), and adjusted scores have not yet been released by ALSPAC.

CDI expressive vocabulary scores have high reliability and validity, showing correlations with children’s laboratory task performance of over 0.70 [33,34]. The correlation of parent-reported receptive vocabulary with children’s language scores in a laboratory setting was moderate, with a correlation of 0.55, but still meaningful [34]. In total, N≤6,524 children (Table 1) had vocabulary scores and genome-wide genetic data available for analyses.

#### Mid-childhood measures

For the selection of mid-childhood measures, we build on our previous work identifying genetic links between vocabulary at 38 months and thirteen mid-childhood/adolescent literacy and cognitive measures [15]. As it is not possible, due to computational constraints, to study longitudinal genetic architectures of early-life vocabulary measures in combination with a wide spectrum of mid-childhood language, literacy and cognitive abilities, we selected three mid-childhood measures that are representative of previously observed developmental association patterns [15] (N≤5,296; S1 Table). The studied mid-childhood measures included a measure of combined reading accuracy and comprehension at 7 years, assessed using the Wechsler Objective Reading Dimensions (WORD)[35], as well as both VIQ and PIQ assessed at 8 years using the Wechsler Intelligence Scale for Children (WISC-III)[36]. Detailed descriptions, including validity and reliability, of each measure are available in the Supporting Information (S2 Text).

#### Phenotype transformation

All early-life vocabulary and mid-childhood measures were adjusted for sex, age (except for VIQ and PIQ as they were derived using age-specific norms), and the first two principal components (adjusting for subtle differences in ancestry [37]). In addition, early-life vocabulary measures were adjusted for age squared, as vocabulary develops rapidly during early childhood [38]. After covariate adjustment, all scores were rank-transformed (rank-based inverse normal transformation of residuals, van der Waerden), a useful data transformation for the analysis of extremely non-normal data with large sample sizes, aiming to increase power [39]. Phenotypic correlations between early-life vocabulary measures were estimated using untransformed (Spearman rank-correlation) and rank-transformed (Pearson correlation) scores respectively, and patterns were largely unaffected by trait transformation (S1 Fig). Phenotypic correlations between early-life vocabulary and mid-childhood reading, VIQ and PIQ measures were estimated using rank-transformed (Pearson correlation) scores only.

### Genome-wide complex trait analysis

Total GCTA-SNP-h^2^ was estimated using Genome-based restricted maximum likelihood (GREML) analyses [40,41], as implemented in GCTA software (https://cnsgenomics.com/software/gcta/) [21], based on a GRM including directly genotyped SNPs only and unrelated individuals (genomic relatedness<0.05). Measures with little evidence for GCTA-SNP-h^2^ (*P*>0.05) were excluded from further analyses.

Bivariate GREML [41] was applied to estimate bivariate genetic correlations among early-life vocabulary measures and between early-life vocabulary and mid-childhood reading, VIQ and PIQ measures.

### Multivariate genetic analyses

To study the genetic architecture of vocabulary in a developmental context and to identify multivariate genetic variance structures underlying bivariate genetic correlation patterns, we used Genetic-relationship-matrix Structural Equation Models (GSEMs)[20]. This is a multivariate structural equation modelling technique, which combines multivariate analysis methodologies established in twin research [42,43] with estimates of genetic relationships between unrelated individuals, as captured by genome-wide genetic markers [20] (S3 Text). Specifically, GSEMs dissect the phenotypic covariance structure into one or more additive genetic factors (A), capturing genetic variance tagged by common genotyped SNPs, as well as one or more residual factors (E) that resemble the residual variance. Latter may contain untagged genetic variation, measurement error and environmental influences. Here, multivariate GSEMs were fitted to the data using a Cholesky decomposition model (S2 Fig), where the phenotypic variance was decomposed into as many latent genetic and residuals factors as there are observed variables, without any restrictions on the structure [44] (S3 Text). This saturated model enables a hypothesis-free examination of longitudinal relationships between measures, while allowing for both shared (i.e. across age and/or ability) and unique (i.e. age- and ability-specific) genetic influences. Structural models were based on all available observations across individuals and thus allow for missing data (saturated model; R:gsem library, version 0.1.5, https://gitlab.gwdg.de/beate.stpourcain/gsem). Genetic relationships between individuals were assessed with GRMs, including directly genotyped SNPs only, as implemented in GCTA software [21].

## Supporting information

S1 Text ALSPAC description. (DOCX)

S2 Text Mid-childhood ALSPAC measures. (DOCX)

S3 Text Genetic-relatedness-matrix Structural equation modelling. (DOCX)

S4 Text Factorial co-heritability. (DOCX)

S5 Text Bivariate heritability. (DOCX)

S1 Table Mid-childhood measures in the Avon Longitudinal Study of Parents and Children. (DOCX)

S2 Table Distributional properties of early-life vocabulary and mid-childhood reading and cognitive skills. (DOCX)

S3 Table SNP heritability estimates. (DOCX)

S4 Table Standardised path coefficients and variance explained for early-life vocabulary measures. (DOCX)

S5 Table Factorial co-heritability for early-life vocabulary measures. (DOCX)

S6 Table Bivariate heritability for early-life vocabulary measures. (DOCX)

S7 Table Standardised path coefficients and variance explained for early-life vocabulary and mid-childhood reading accuracy/comprehension. (DOCX)

S8 Table Standardised path coefficients and variance explained for early-life vocabulary and mid-childhood verbal intelligence. (DOCX)

S9 Table Standardised path coefficients and variance explained for early-life vocabulary and mid-childhood performance intelligence. (DOCX)

S10 Table Factorial co-heritability for genetic factors contributing to mid-childhood reading, verbal intelligence and performance intelligence. (DOCX)

S11 Table Bivariate heritability for early-life vocabulary measures and mid-childhood reading, verbal intelligence and performance intelligence. (DOCX)

S1 Fig Phenotypic correlations among early-life vocabulary measures. Phenotypic correlations among untransformed (lower triangle) and rank-transformed (upper triangle) measures with sufficient evidence for SNP-h^2^ (*P*>0.05) were estimated with Spearman’s rank and Pearson correlation coefficients respectively. All phenotypic correlation coefficients passed the significance threshold of *P*<0.05. Abbreviations: CDI, Communicative Development Inventory; m, months; voc, vocabulary.
(TIF)

S2 Fig Path diagram for a trivariate trait. The variance/covariance structure of multivariate trait consisting of three standardised measures P1, P2 and P3 can be described using a Cholesky decomposition consisting of three genetic factors (A1, A2 and A3) and three residual factors (E1, E2 and E3), shown here with genetic and residual factor loadings (path coefficients). The observed phenotypic measures are represented by squares, while all latent genetic and residual factors are represented by a circle. Single headed arrows (’paths’) denote causal relationships between variables and are shown for genetic factor loadings (a) and residual factor loadings (e). Note that the variance of latent variables is constrained to unit variance, this is omitted from the diagrams to improve clarity.
(TIF)
