# Supplementary material for: The developmental genetic architecture of vocabulary skills during the first three years of life: Capturing emerging associations with later-life reading and cognition
Source: PLoS Genet. 2021 Feb 12;17(2):e1009144. doi: 10.1371/journal.pgen.1009144 (PMC7880480; doi:10.1371/journal.pgen.1009144)
Supplement: S2 Text — (DOCX) [file pgen.1009144.s002.docx]

## **S2 Text. Mid-childhood ALSPAC measures**

**Reading accuracy and comprehension age 7 (WORD).** Decoding and word reading were assessed using the basic reading subtest of the Wechsler Objective Reading Dimensions (WORD)[1], including both pictures and words. This test has reliability and internal consistency coefficients ≥0.9. Its validity is also high, with an inter-correlation of 0.82 with the word-reading test from the Differential Ability Scale [2]. In short, the child was shown a series of four pictures, and each picture had four short words underneath it. For each picture, the child was asked to point to the word underneath that had the same beginning or ending sound as the picture. This was followed by a series of three pictures that all had four words beneath them starting with the same letter as the picture. This time the child was asked to point to the word that correctly named the picture. Finally, the child was presented with a series of 48 unconnected words, which increased in difficulty, and asked to read them aloud. The task was stopped if the child made six consecutive errors. A reading accuracy and comprehension score, that had a maximum score of 50, was computed as the sum of the number of items the child read/responded to correctly.

**Verbal intelligence age 8 (WISC-III).** To assess verbal intelligence the child was assessed using a short form of the Wechsler Intelligence Scale for Children (WISC-III)[3]. The WISC-III comprises ten subtests and alternate items were administered for all subtests, but the coding subtest. The information, similarities, arithmetic, vocabulary, and comprehension subtests were used to create a score indicating verbal intelligence. The WISC-III verbal intelligence score has high test-retest correlations, ranging between 0.90 and 0.94, dependent on the age at assessment and the duration of the test-retest interval [4]. Correlations with the Kaufman Brief Intelligence Test and the Stanford-Binet IV suggest good construct validity, with estimates of 0.79 and 0.69 respectively [4]. After the calculation of raw scores based on the items used in the alternate item form of the WISC-III, total age-scaled scores for the verbal scale were calculated according to the look-up tables in the WISC-III manual. The maximum VIQ score is 160 and all scores were pro-rated.

**Performance intelligence age 8 (WISC-III).** Performance intelligence was assessed using a short form of the Wechsler Intelligence Scale for Children (WISC-III)[3], including alternate items for all subtests, with the exception of the coding subtest. The five performance subtests of the WISC-III were used to create a score indicating performance intelligence: picture completion, coding, picture arrangement, block design and object assembly. The WISC-III performance intelligence quotient (PIQ) score has a correlation of 0.59 with the non-verbal score measured using the Otis-Lennon School Ability Test was 0.59 [5]. It has high reliability, with test-retest correlations of 0.89 [6]. Raw PIQ scores were calculated based on the items used in the alternate item form of the WISC-III and had a maximum PIQ score of 160. Next, total age-scaled scores for the performance scale were calculated according to the look-up tables in the WISC-III manual. All scores were pro-rated.

References

1. WORD, Wechsler Objective Reading Dimensions Manual. Psychological Corporation; 1993.

2. Elliott CD, Salerno JD, Dumont R, Willis JO. The Differential Ability Scales—Second Edition. Contemporary intellectual assessment: Theories, tests, and issues, 4th ed. New York, NY, US: The Guilford Press; 2018. pp. 360–382.

3. Wechsler D, Golombok S, Rust J. WISC-III UK Wechsler Intelligence Scale for Children – Third Edition UK Manual. Sidcup, UK: The Psychological Corporation; 1992.

4. Sattler JM. Assessment of children: Cognitive applications, 4th ed. La Mesa, CA, US: Jerome M Sattler Publisher; 2001.

5. Guilmette TJ, Kennedy ML, Queally PT. A Comparison of the WISC-III and the Otis-Lennon School Ability Test with Students Referred for Learning Disabilities. Journal of Psychoeducational Assessment. 2001;19: 239–244. doi:10.1177/073428290101900304

6. Canivez GL, Watkins MW. Long-term stability of the Wechsler Intelligence Scale for Children—Third Edition. Psychological Assessment. 1998;10: 285–291. doi:10.1037/1040-3590.10.3.285
