# Supplementary material for: The developmental genetic architecture of vocabulary skills during the first three years of life: Capturing emerging associations with later-life reading and cognition
Source: PLoS Genet. 2021 Feb 12;17(2):e1009144. doi: 10.1371/journal.pgen.1009144 (PMC7880480; doi:10.1371/journal.pgen.1009144)
Supplement: S3 Text — (DOCX) [file pgen.1009144.s003.docx]

## **S3 Text. Genetic-relatedness-matrix Structural equation modelling**

A Cholesky decomposition [1] describes a multivariate trait P with phenotypic measurements t, resulting in a range of measures (P_1_,P_2_,...,P_t_). The first measure (P_1_) can be influenced by a latent genetic factor (A_1_) that may also explain variance in the remaining measures (P_2_,...,P_t_). The second measure (P_2_) can also be influenced by a second latent genetic factor (A_2_) that is independent of A_1_ and captures additional variation. As the first latent genetic factor, the second latent genetic factor may also capture variance in all other measures (P_3_,...,P_t_). The final measure (P_t_) can thus be influenced by latent genetic factors (A_1_,...,A_t-1_), but also a latent genetic factor A_t_. This latter genetic factor (A_t_) is independent of previous latent genetic factors (A_1_,...,A_t-1_) and does not explain variance within any of the previous measures (P_1_,...,P_t-1_)[2].

Based on the factor model, the expected phenotypic covariance matrix $\Sigma$ for Z-standardised traits is:

(1)

with a lower triangular matrix of genetic factor loadings $\Lambda$, a diagonal matrix of latent genetic factor variances $\Phi$ (standardised to unit variance), a lower triangular matrix of residual factor loadings and a diagonal matrix of latent residual factor variances $\Theta$. Both $\Phi$ and $\Theta$were standardised to unit variance, such that they represent an identity matrix ***I*** [3].

For example, for a Cholesky decomposition of three measures (P_1_, P_2_ and P_3_), assuming three latent genetic factors (A_1_, A_2_ and A_3_) and three residual factors (E_1_, E_2_ and E_3_) this translates into the following expected phenotypic covariance matrix:

$\Sigma=\left[ \begin{matrix} \sigma_{p1}^{2} & \sigma_{p12} & \sigma_{p13} \\ \sigma_{p21} & \sigma_{p2}^{2} & \sigma_{p23} \\ \sigma_{p31} & \sigma_{p32} & \sigma_{p3}^{2} \end{matrix} \right]$ (2)

with phenotypic variances $\sigma_{p1}^{2}$,$\sigma_{p2}^{2}$and $\sigma_{p3}^{2}$, phenotypic covariances $\sigma_{p12}$, $\sigma_{p13}$ and $\sigma_{p23}$, and the relevant matrices

$\Lambda=\left[\begin{matrix} a_{11} & 0 & 0 \\ a_{21} & a_{22} & 0 \\ a_{31} & a_{32} & a_{33} \end{matrix}\right], \Phi= \left[\begin{matrix} 1 & 0 & 0 \\ 0 & 1 & 0 \\ 0 & 0 & 1 \end{matrix}\right], \Gamma= \left[\begin{matrix} e_{11} & 0 & 0 \\ e_{21} & e_{22} & 0 \\ e_{31} & e_{32} & e_{33} \end{matrix}\right], \Theta= \left[\begin{matrix} 1 & 0 & 0 \\ 0 & 1 & 0 \\ 0 & 0 & 1 \end{matrix}\right]$ (3)

with genetic factor loadings a and residual factor loadings e.

Factor loadings, also known as path coefficients, were annotated with *a* or *e* followed by two numbers to indicate the specific path. In this notation, the first number indicates the direction of effect (the measure to which the path goes) and the second number indicates the origin of the effect [2]. For example, a_21_ indicates the genetic factor loading for the path originating from A_1_ and affecting P_2_.

A Cholesky decomposition of three standardised measures (see above), can be visualised using a path diagram (S2 Fig). The expected phenotypic variances and covariances can be expressed as follows:

$\sigma_{p1}^{2}=a_{11}^{2}+e_{11}^{2}= 1$ (4)

$\sigma_{p2}^{2}=(a_{21}^{2}+a_{22}^{2})+(e_{21}^{2}+e_{22}^{2})= 1$ (5)

$\sigma_{p3}^{2}=(a_{31}^{2}+a_{32}^{2}+a_{33}^{2})+(e_{31}^{2}+e_{32}^{2}+e_{33}^{2})= 1$ (6)

$\sigma_{p12}=a_{11}a_{21}+e_{11}e_{21}$ (7)

$\sigma_{p13}=a_{11}a_{31}+e_{11}e_{31}$ (8)

$\sigma_{p23}=a_{31}a_{21}+a_{32}a_{22}+ e_{31}e_{21}+e_{32}e_{22}$ (9)

The variance of the latent genetic and residual factors has been standardised to unit variance and is not shown.

Bivariate genetic correlation estimates (ranging from -1 to 1) reflect the extent to which two measures share genetic factors and can be derived using estimated genetic variances and covariances [4] according to:

$r_{g}=\frac{\sigma_{g12}}{\sqrt{\sigma_{g1}^{2}\sigma_{g2}^{2}}}$ (10)

with genetic covariance $\sigma_{g12}$between measures P_1_ and P_2_, and the genetic variances $\sigma_{g1}^{2}$ and $\sigma_{g2}^{2}$.

References

1. Cherny SS. Cholesky Decomposition. Encyclopedia of Statistics in Behavioral Science. John Wiley & Sons, Ltd; 2005.

2. Neale M, Maes HHM. Methodology for genetic studies of twins and families. Dordrecht: Kluwer Academic Publishers; 2004.

3. Martin NG, Eaves LJ. The genetical analysis of covariance structure. Heredity (Edinb). 1977;38: 79–95.

4. Falconer PDS, Mackay PTFC. Introduction to Quantitative Genetics. 4 edition. Essex, England: Longman; 1995.
