# Supplementary material for: The developmental genetic architecture of vocabulary skills during the first three years of life: Capturing emerging associations with later-life reading and cognition
Source: PLoS Genet. 2021 Feb 12;17(2):e1009144. doi: 10.1371/journal.pgen.1009144 (PMC7880480; doi:10.1371/journal.pgen.1009144)
Supplement: S4 Text — (DOCX) [file pgen.1009144.s004.docx]

## **S4 Text. Factorial co-heritability**

Factorial co-heritability was estimated to quantify the relative contribution of a genetic factor to the total genetic variance of a phenotype, using the gsem package (R:gsem library, version 0.1.5). We derived the factorial co-heritability (f_g_^2^) according to:

(11)


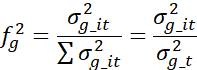


with the genetic variance of genetic factor i contributing to trait t σ^2^_g_it_  and the total genetic variance of trait t σ^2^_g_t_. Estimates were derived using standardised path coefficients, corresponding standard errors (SEs) using the Delta method, and *P*-values approximated with a Wald test.
