# Supplementary material for: The developmental genetic architecture of vocabulary skills during the first three years of life: Capturing emerging associations with later-life reading and cognition
Source: PLoS Genet. 2021 Feb 12;17(2):e1009144. doi: 10.1371/journal.pgen.1009144 (PMC7880480; doi:10.1371/journal.pgen.1009144)
Supplement: S5 Text — (DOCX) [file pgen.1009144.s005.docx]

## **S5 Text. Bivariate heritability**

The proportion of phenotypic covariance between two traits that is accounted for by the genetic covariance was expressed as bivariate heritability [1], as incorporated in the gsem package (R:gsem library, version 0.1.5). It was estimated based on unstandardised path coefficients and the phenotypic covariance estimated for rank-transformed measures. Corresponding SEs were approximated by dividing the SE of the genetic covariance by the phenotypic covariance, based on the assumption that the SE of the phenotypic covariance is small. *P*-values were calculated using a Wald-test assuming normality.

References

1. Janssens MJJ. Co-heritability: Its relation to correlated response, linkage, and pleiotropy in cases of polygenic inheritance. Euphytica. 1979;28: 601–608. doi:10.1007/BF00038926
