# Supplementary material for: The developmental genetic architecture of vocabulary skills during the first three years of life: Capturing emerging associations with later-life reading and cognition
Source: PLoS Genet. 2021 Feb 12;17(2):e1009144. doi: 10.1371/journal.pgen.1009144 (PMC7880480; doi:10.1371/journal.pgen.1009144)
Supplement: S1 Table — (DOCX) [file pgen.1009144.s006.docx]

## **S1 Table. Mid-childhood measures in ALSPAC**

| **Measure** | **Psychological instrument** | **Mean score (SE)** | **Mean age (SE) in years** | **N**  **(%males)** |
| --- | --- | --- | --- | --- |
| Reading accuracy and comprehension | WORD | 28.52 (9.25) | 7.53 (0.31) | 5,723 (50.9) |
| Verbal intelligence^a^ | WISC-III | 108.04 (16.74) | 8.64 (0.31) | 5,305 (49.9) |
| Performance intelligence^a^ | WISC-III | 100.24 (16.95) | 8.64 (0.31) | 5,296 (49.9) |

a. Scores were derived using age norms and adjusted for sex and principal components only before transformation (see Methods).

Representative of previous findings [1] reading skills and verbal intelligence during mid-childhood (7-8 year) were selected from ALSPAC. To compare findings between verbal and non-verbal cognitive processes, mid-childhood performance intelligence was also studied. Skills were assessed using standardised instruments and studied in independent ALSPAC participants only (genetic relatedness<0.05).

Abbreviations: ALSPAC, Avon Longitudinal Study of Parents and Children; WISC-III, Wechsler Intelligence Scale for Children III; WORD, Wechsler Objective Reading Dimension

References

1. Verhoef E, Shapland CY, Fisher SE, Dale PS, Pourcain BS. The developmental origins of genetic factors influencing language and literacy: Associations with early-childhood vocabulary. Journal of Child Psychology and Psychiatry. 2020. doi:10.1111/jcpp.13327
