# Supplementary material for: The developmental genetic architecture of vocabulary skills during the first three years of life: Capturing emerging associations with later-life reading and cognition
Source: PLoS Genet. 2021 Feb 12;17(2):e1009144. doi: 10.1371/journal.pgen.1009144 (PMC7880480; doi:10.1371/journal.pgen.1009144)
Supplement: S2 Table — (DOCX) [file pgen.1009144.s007.docx]

## **S2 Table: Distributional properties of early-life vocabulary and mid-childhood reading and cognitive skills**

| **Measure** | **Mean score (SE)** | **Median score** | **Skewness** | **Kurtosis** | **N** |
| --- | --- | --- | --- | --- | --- |
| Expressive vocabulary 15m (CDI) | 14.29 (17.76) | 8 | 2.38 | 7.38 | 6,524 |
| Receptive vocabulary 15m (CDI) | 75.85(31.78) | 74 | -0.11 | -0.72 | 6,524 |
| Expressive vocabulary 24m (CDI) | 64.21 (35.11) | 65 | -0.04 | -1.12 | 6,014 |
| Expressive vocabulary 38m (CDI) | 113.33 (17.44) | 120 | -3.43 | 14.53 | 6,092 |
| Receptive vocabulary 38m (CDI) | 109.75 (23.75) | 118 | -3.25 | 11.01 | 6,092 |
| Reading a/c 7 (WORD) | 28.52 (9.25) | 29 | -0.25 | -0.50 | 5,723 |
| VIQ 8 (WISC-III) | 108.04 (16.74) | 107 | -0.01 | -0.20 | 5,305 |
| PIQ 8 (WISC-III) | 100.24 (16.95) | 99 | 0.002 | -0.30 | 5,296 |

Distributional properties, including mean, median, skewness and kurtosis, were derived for unadjusted early-life vocabulary measures, as well as unadjusted mid-childhood reading and cognitive skills.

Abbreviations: a, accuracy; c, comprehension; CDI, Communicative Development Inventory; PIQ, verbal intelligence quotient; VIQ, verbal intelligence quotient; WISC-III, Wechsler Intelligence Scale for Children III; WORD, Wechsler Objective Reading Dimension
