# Supplementary material for: The developmental genetic architecture of vocabulary skills during the first three years of life: Capturing emerging associations with later-life reading and cognition
Source: PLoS Genet. 2021 Feb 12;17(2):e1009144. doi: 10.1371/journal.pgen.1009144 (PMC7880480; doi:10.1371/journal.pgen.1009144)
Supplement: S3 Table — (DOCX) [file pgen.1009144.s008.docx]

**S3 Table. SNP heritability estimates**

| **Measure** | **N** | **GCTA-h^2^(SE)** | **GSEM-h^2^(SE)** |
| --- | --- | --- | --- |
| Expressive vocabulary 15m (CDI) | 6,524 | 0.11(0.05) | 0.11(0.05)^b^ |
| Receptive vocabulary 15m (CDI)^a^ | 6,524 | 0.08(0.05) | NA |
| Expressive vocabulary 24m (CDI) | 6,014 | 0.16(0.06) | 0.15(0.06)^b^ |
| Expressive vocabulary 38m (CDI) | 6,092 | 0.18(0.06) | 0.18(0.06)^b^ |
| Receptive vocabulary 38m (CDI) | 6,092 | 0.12(0.06) | 0.12(0.05)^b^ |
| Reading a/c 7 (WORD) | 5,723 | 0.42(0.06) | 0.41(0.06) |
| VIQ 8 (WISC-III) | 5,305 | 0.54(0.07) | 0.54(0.06) |
| PIQ 8 (WISC-III) | 5,296 | 0.26(0.07) | 0.27(0.06) |

a. Due to limited evidence (*P*>0.05) for SNP-h^2^ of receptive vocabulary assessed at 15 months using GCTA, this trait was excluded from further analyses.

b. GSEM-h^2^ estimates as observed in the GSEM model with reading accuracy/comprehension at 7 years.

SNP-heritability estimates were estimated based on rank-transformed scores, directly genotyped SNPs and individuals with a genetic relationship of <0.05 using Restricted Maximum Likelihood (REML) analyses as implemented in genome-wide complex trait analysis (GCTA) software. SNP-heritability estimates based on Genetic-relationship-matrix Structural Equation modelling (GSEM) were extracted for comparison.

Abbreviations: a, accuracy; c, comprehension; CDI, Communicative Development Inventory; GCTA, genome-wide complex trait analysis; GSEM, Genetic-relationship-matrix Structural Equation modelling; h^2^, heritability; PIQ, verbal intelligence quotient; VIQ, verbal intelligence quotient; WISC-III, Wechsler Intelligence Scale for Children III; WORD, Wechsler Objective Reading Dimension
