# Supplementary material for: The developmental genetic architecture of vocabulary skills during the first three years of life: Capturing emerging associations with later-life reading and cognition
Source: PLoS Genet. 2021 Feb 12;17(2):e1009144. doi: 10.1371/journal.pgen.1009144 (PMC7880480; doi:10.1371/journal.pgen.1009144)
Supplement: S4 Table — (DOCX) [file pgen.1009144.s009.docx]

**S4 Table. Standardised path coefficients and variance explained for early-life vocabulary measures**

| **Path** | **Standardised path coefficient** | | **Standardised variance explained (%)** |
| --- | --- | --- | --- |
|  | **Estimate (SE)** | ***P*** | **Estimate (SE)** |
| **a_11_** | 0.33(0.08) | 2x10^-5^ | 10.6(5.0) |
| **a_21_** | 0.21(0.10) | 0.04 | 4.6(4.4) |
| **a_31_** | 0.15(0.11) | 0.18 | 2.2(3.3) |
| **a_41_** | -3x10^-3^(0.11) | 0.98 | 7x10^-4^(0.1) |
| **a_22_** | 0.32(0.06) | 4x10^-7^ | 10.1(4.0) |
| **a_32_** | 0.27(0.09) | 0.005 | 7.1(5.0) |
| **a_42_** | 0.33(0.08) | 4x10^-5^ | 11.0(5.3) |
| **a_33_** | 0.29(0.08) | 0.001 | 8.2(4.9) |
| **a_43_** | 0.12(0.12) | 0.35 | 1.4(2.9) |
| **a_44_** | 1x10^-4^(0.24) | 1.00 | 1x10^-6^(0.005) |
| **e_11_** | 0.95(0.03) | <1x10^-10^ | 89.4(5.0) |
| **e_21_** | 0.49(0.04) | <1x10^-10^ | 24.4(3.7) |
| **e_31_** | 0.22(0.04) | 6x10^-8^ | 4.8(1.8) |
| **e_41_** | 0.23(0.04) | 3x10^-9^ | 5.2(1.8) |
| **e_22_** | -0.78(0.03) | <1x10^-10^ | 60.9(4.0) |
| **e_32_** | -0.33(0.04) | <1x10^-10^ | 10.6(2.7) |
| **e_34_** | -0.22(0.04) | 3x10^-8^ | 4.9(1.8) |
| **e_33_** | 0.82(0.03) | <1x10^-10^ | 67.0(4.4) |
| **e_43_** | 0.47(0.04) | <1x10^-10^ | 22.3(3.3) |
| **e_44_** | 0.74(0.02) | <1x10^-10^ | 55.3(2.9) |

Genetic-relationship matrix structural equation modelling (GSEM) of rank-transformed early-life vocabulary scores (expressive vocabulary at 15, 24 and 38 months of age and receptive vocabulary at 38 months of age) based on all available observations for children across development (N≤6,524; Cholesky decomposition model). A visual representation is provided in Fig 2.
