# Supplementary material for: The developmental genetic architecture of vocabulary skills during the first three years of life: Capturing emerging associations with later-life reading and cognition
Source: PLoS Genet. 2021 Feb 12;17(2):e1009144. doi: 10.1371/journal.pgen.1009144 (PMC7880480; doi:10.1371/journal.pgen.1009144)
Supplement: S5 Table — (DOCX) [file pgen.1009144.s010.docx]

**S5 Table. Factorial co-heritability for early-life vocabulary measures**

| **Path** | **Factorial co-heritability (%)** | |
| --- | --- | --- |
|  | **Estimate (SE)** | ***P*** |
| **a_11_** | 100.0 (0.0) | <1x10^-10^ |
| **a_21_** | 31.2 (23.4) | 0.18 |
| **a_31_** | 12.7 (18.1) | 0.48 |
| **a_41_** | 0.005 (0.5) | 0.99 |
| **a_22_** | 68.8 (23.4) | 0.003 |
| **a_32_** | 40.3 (25.6) | 0.12 |
| **a_42_** | 88.9 (23.1) | 1x10^-4^ |
| **a_33_** | 47.0 (25.1) | 0.06 |
| **a_43_** | 11.1 (23.0) | 0.63 |
| **a_44_** | 8x10^-6^(0.04) | 1.00 |

Factorial co-heritability reflects the proportion of total SNP-h^2^ estimated for a trait explained by a specific genetic factor. SEs were derived using the Delta method and *P*-values based on a Wald-test assuming normality (S4 Text). For example, the factorial co-heritability of a_42_ was estimated as a_42_*a_42_ / (a_41_*a_41_ + a_42_*a_42_ + a_43_*a_43_ + a_44_*a_44_) *100% and implies that genetic factor A2 explains 88.9%(SE=23.1%) of the total SNP-h^2^ estimated for receptive vocabulary at 38 months (see Fig 2).
