# Supplementary material for: The developmental genetic architecture of vocabulary skills during the first three years of life: Capturing emerging associations with later-life reading and cognition
Source: PLoS Genet. 2021 Feb 12;17(2):e1009144. doi: 10.1371/journal.pgen.1009144 (PMC7880480; doi:10.1371/journal.pgen.1009144)
Supplement: S6 Table — (DOCX) [file pgen.1009144.s011.docx]

**S6 Table. Bivariate heritability for early-life vocabulary measures**

|  | Expressive voc 15m (CDI) | Expressive voc 24m (CDI) | Expressive voc 38m (CDI) | Receptive voc 38m (CDI) |
| --- | --- | --- | --- | --- |
| Expressive voc 15m (CDI) |  |  |  |  |
| Expressive voc 24m (CDI) | 0.13  (0.08) |  |  |  |
| Expressive voc 38m (CDI) | 0.20  (0.16) | 0.25*  (0.09) |  |  |
| Receptive voc 38m (CDI) | -0.004  (0.17) | 0.28*  (0.11) | 0.19*  (0.07) |  |

* Bivariate heritability estimates passing a significance threshold of *P*<0.05.

Bivariate heritability reflects the proportion of the phenotypic covariance between two traits that is accounted for by the genetic covariance. Standard errors (SEs) are shown in brackets and were approximated by the SE of the genetic covariance divided by the phenotypic covariance (as the SE of the phenotypic covariance is small). *P*-values are based on a Wald-test, assuming normality.

Abbreviations: CDI, communicative development inventory; m, months; voc, vocabulary
