# Supplementary material for: The developmental genetic architecture of vocabulary skills during the first three years of life: Capturing emerging associations with later-life reading and cognition
Source: PLoS Genet. 2021 Feb 12;17(2):e1009144. doi: 10.1371/journal.pgen.1009144 (PMC7880480; doi:10.1371/journal.pgen.1009144)
Supplement: S7 Table — (DOCX) [file pgen.1009144.s012.docx]

**S7 Table. Standardised path coefficients and variance explained for early-life vocabulary and mid-childhood reading accuracy/comprehension**

| **Path** | **Standardised path coefficient** | | **Standardised variance explained (%)** |
| --- | --- | --- | --- |
|  | **Estimate (SE)** | ***P*** | **Estimate (SE)** |
| **a_11_** | 0.33(0.08) | 3x10^-5^ | 10.6(5.1) |
| **a_21_** | 0.22(0.10) | 0.04 | 4.7(4.5) |
| **a_31_** | 0.15(0.11) | 0.19 | 2.1(3.3) |
| **a_41_** | 0.01(0.11) | 0.93 | 0.01(0.2) |
| **a_51_** | -0.08(0.12) | 0.50 | 0.7(2.1) |
| **a_22_** | 0.32(0.06) | 1x10^-6^ | 10.0(4.1) |
| **a_32_** | 0.26(0.10) | 0.01 | 7.0(5.1) |
| **a_42_** | 0.30(0.09) | 3x10^-4^ | 9.3(5.2) |
| **a_52_** | 0.25(0.12) | 0.04 | 6.4(6.2) |
| **a_33_** | 0.29(0.09) | 0.001 | 8.1(4.9) |
| **a_43_** | 0.13(0.11) | 0.24 | 1.7(3.0) |
| **a_53_** | 0.02(0.16) | 0.93 | 0.02(0.5) |
| **a_44_** | 0.15(0.06) | 0.02 | 2.1(1.9) |
| **a_54_** | 0.57(0.07) | <1x10^-10^ | 33.0(8.2) |
| **a_55_** | -3x10^-4^(0.58) | 1.00 | 9x10^-6^(0.03) |
| **e_11_** | 0.95(0.03) | <1x10^-10^ | 89.4(5.1) |
| **e_21_** | 0.49(0.04) | <1x10^-10^ | 24.4(3.8) |
| **e_31_** | 0.22(0.04) | 5x10^-8^ | 4.8(1.8) |
| **e_41_** | 0.22(0.04) | 2x10^-9^ | 5.0(1.7) |
| **e_51_** | 0.15(0.04) | 2x10^-4^ | 2.3(1.2) |
| **e_22_** | -0.28(0.03) | <1x10^-10^ | 61.0(4.1) |
| **e_32_** | -0.33(0.04) | <1x10^-10^ | 10.6(2.7) |
| **e_42_** | -0.23(0.04) | 4x10^-9^ | 5.3(1.8) |
| **e_52_** | -0.11(0.04) | 0.01 | 1.2(1.0) |
| **e_33_** | -0.82(0.03) | <1x10^-10^ | 67.2(4.4) |
| **e_43_** | -0.47(0.03 | <1x10^-10^ | 22.1(3.1) |
| **e_53_** | -0.06(0.04) | 0.18 | 0.3(0.5) |
| **e_44_** | 0.74(0.02) | <1x10^-10^ | 54.4(2.3) |
| **e_54_** | -0.07(0.04) | 0.08 | 0.5(0.5) |
| **e_55_** | 0.75(0.04) | <1x10^-10^ | 55.5(5.6) |

Genetic-relationship matrix structural equation modelling (GSEM) of rank-transformed early-life vocabulary scores (15, 24 and 38 months of age) in combination with rank-transformed mid-childhood reading accuracy/comprehension at 7 years, based on all available observations for children across development (N≤6,524). A visual representation is provided in Figs 4a and 4b.
