# Supplementary material for: The developmental genetic architecture of vocabulary skills during the first three years of life: Capturing emerging associations with later-life reading and cognition
Source: PLoS Genet. 2021 Feb 12;17(2):e1009144. doi: 10.1371/journal.pgen.1009144 (PMC7880480; doi:10.1371/journal.pgen.1009144)
Supplement: S8 Table — (DOCX) [file pgen.1009144.s013.docx]

**S8 Table. Standardised path coefficients and variance explained for early-life vocabulary and mid-childhood verbal intelligence**

| **Path** | **Standardised path coefficient** | | **Standardised variance explained (%)** |
| --- | --- | --- | --- |
|  | **Estimate (SE)** | ***P*** | **Estimate (SE)** |
| **a_11_** | -0.33(0.08) | 2x10^-5^ | 10.8(5.0) |
| **a_21_** | -0.22(0.10) | 0.03 | 4.8(4.6) |
| **a_31_** | -0.15(0.11) | 0.18 | 2.2(3.3) |
| **a_41_** | -0.01(0.10) | 0.39 | 0.02(0.3) |
| **a_51_** | 0.05(0.13) | 0.70 | 0.2(1.2) |
| **a_22_** | 0.32(0.07) | 1x10^-6^ | 10.1(4.1) |
| **a_32_** | 0.26(0.10) | 0.008 | 6.6(5.0) |
| **a_42_** | 0.31(0.09) | 3x10^-4^ | 9.7(5.3) |
| **a_52_** | 0.42(0.13) | 0.001 | 17.9(11.1) |
| **a_33_** | -0.29(0.08) | 4x10^-4^ | 8.5(4.8) |
| **a_43_** | -0.14(0.11) | 0.22 | 1.9(3.1) |
| **a_53_** | -0.02(0.19) | 0.91 | 0.1(0.8) |
| **a_44_** | 0.15(0.07) | 0.04 | 2.2(2.1) |
| **a_54_** | 0.60(0.10) | 3x10^-10^ | 36.1(11.5) |
| **a_55_** | 4x10^-4^(0.56) | 1.00 | 2x10^-5^(0.1) |
| **e_11_** | -0.94(0.03) | <1x10^-10^ | 89.2(5.0) |
| **e_21_** | -0.49(0.04) | <1x10^-10^ | 24.2(3.8) |
| **e_31_** | -0.22(0.04) | 6x10^-8^ | 4.8(1.8) |
| **e_41_** | -0.22(0.04) | 2x10^-9^ | 4.9(1.6) |
| **e_51_** | -0.11(0.04) | 0.01 | 1.3(1.0) |
| **e_22_** | 0.78(0.03) | <1x10^-10^ | 60.8(4.1) |
| **e_32_** | 0.33(0.04) | <1x10^-10^ | 10.8(2.7) |
| **e_42_** | 0.23(0.04) | 6x10^-9^ | 5.1(1.8) |
| **e_52_** | 0.08(0.05) | 0.08 | 0.7(0.8) |
| **e_33_** | 0.82(0.03) | <1x10^-10^ | 67.1(4.4) |
| **e_43_** | 0.47(0.03) | <1x10^-10^ | 21.9(3.1) |
| **e_53_** | 0.03(0.05) | 0.45 | 0.1(0.3) |
| **e_44_** | -0.74(0.02) | <1x10^-10^ | 54.2(2.3) |
| **e_54_** | 0.02(0.04) | 0.59 | 0.05(0.2) |
| **e_55_** | -0.66(0.05) | <1x10^-10^ | 43.6(6.0) |

Genetic-relationship matrix structural equation modelling (GSEM) of rank-transformed early-life vocabulary scores (15, 24 and 38 months of age) in combination with rank-transformed mid-childhood verbal intelligence scores at 8 years, based on all available observations for children across development (N≤6,524). A visual representation is provided in Figs 4c and 4d.
