# Supplementary material for: The developmental genetic architecture of vocabulary skills during the first three years of life: Capturing emerging associations with later-life reading and cognition
Source: PLoS Genet. 2021 Feb 12;17(2):e1009144. doi: 10.1371/journal.pgen.1009144 (PMC7880480; doi:10.1371/journal.pgen.1009144)
Supplement: S9 Table — (DOCX) [file pgen.1009144.s014.docx]

**S9 Table. Standardised path coefficients and variance explained for early-life vocabulary and mid-childhood performance intelligence**

| **Path** | **Standardised path coefficient** | | **Standardised variance explained (%)** |
| --- | --- | --- | --- |
|  | **Estimate (SE)** | ***P*** | **Estimate (SE)** |
| **a_11_** | -0.33(0.08) | 2x10^-5^ | 10.8(5.1) |
| **a_21_** | -0.21(0.10) | 0.04 | 4.6(4.4) |
| **a_31_** | -0.15(0.11) | 0.18 | 2.2(3.3) |
| **a_41_** | -0.004(0.11) | 0.97 | 0.001(0.1) |
| **a_51_** | 0.12(0.13) | 0.36 | 1.4(3.0) |
| **a_22_** | -0.32(0.06) | 2x10^-7^ | 10.5(4.0) |
| **a_32_** | -0.25(0.09) | 0.01 | 6.5(4.7) |
| **a_42_** | -0.29(0.08) | 4x10^-4^ | 8.6(4.8) |
| **a_52_** | -0.03(0.12) | 0.78 | 0.1(0.8) |
| **a_33_** | 0.29(0.08) | 3x10^-4^ | 8.6(4.7) |
| **a_43_** | 0.15(0.10) | 0.14 | 2.2(3.1) |
| **a_53_** | 0.09(0.14) | 0.55 | 0.7(2.4) |
| **a_44_** | 0.16(0.07) | 0.02 | 2.5(2.1) |
| **a_54_** | 0.50(0.08) | <1x10^-10^ | 24.7(7.5) |
| **a_55_** | 0.01(0.45) | 0.99 | 0.003(0.4) |
| **e_11_** | -0.94(0.03) | <1x10^-10^ | 89.2(5.1) |
| **e_21_** | -0.49(0.04) | <1x10^-10^ | 24.4(3.8) |
| **e_31_** | -0.22(0.04) | 7x10^-8^ | 4.8(1.8) |
| **e_41_** | -0.23(0.04) | 2x10^-9^ | 5.1(1.7) |
| **e_51_** | -0.09(0.04) | 0.04 | 0.8(0.8) |
| **e_22_** | 0.78(0.03) | <1x10^-10^ | 60.5(4.0) |
| **e_32_** | 0.33(0.04) | <1x10^-10^ | 10.9(2.7) |
| **e_42_** | 0.23(0.04) | 3x10^-9^ | 5.5(1.8) |
| **e_52_** | 0.12(0.05) | 0.01 | 1.5(1.2) |
| **e_33_** | -0.82(0.03) | <1x10^-10^ | 67.0(4.4) |
| **e_43_** | -0.47(0.03) | <1x10^-10^ | 21.9(3.1) |
| **e_53_** | -0.03(0.05) | 0.52 | 0.1(0.3) |
| **e_44_** | 0.74(0.02) | <1x10^-10^ | 54.1(2.6) |
| **e_54_** | -0.01(0.04) | 0.82 | 0.01(0.1) |
| **e_55_** | 0.84(0.04) | <1x10^-10^ | 70.6(6.4) |

Genetic-relationship matrix structural equation modelling (GSEM) of rank-transformed early-life vocabulary scores (15, 24 and 38 months of age) in combination with rank-transformed mid-childhood performance intelligence scores at 8 years, based on all available observations for children across development (N≤6,524). A visual representation is provided in Figs 4e and 4f.
