# Supplementary material for: The developmental genetic architecture of vocabulary skills during the first three years of life: Capturing emerging associations with later-life reading and cognition
Source: PLoS Genet. 2021 Feb 12;17(2):e1009144. doi: 10.1371/journal.pgen.1009144 (PMC7880480; doi:10.1371/journal.pgen.1009144)
Supplement: S10 Table — (DOCX) [file pgen.1009144.s015.docx]

**S10 Table. Factorial co-heritability for genetic factors contributing to mid-childhood reading, verbal intelligence and performance intelligence**

| **Measure** | **Factorial co-heritability (%)** | | | | | | | | | |
| --- | --- | --- | --- | --- | --- | --- | --- | --- | --- | --- |
|  | **Expressive voc 15 months^a^** | | **Expressive voc**  **24 months^b^** | | **Expressive voc**  **38 months^c^** | | **Receptive voc**  **38 months^d^** | | **Mid-childhood LRA^e^** | |
|  | **Estimate (SE)** | ***P*** | **Estimate (SE)** | ***P*** | **Estimate (SE)** | ***P*** | **Estimate (SE)** | ***P*** | **Estimate (SE)** | ***P*** |
| Reading a/c 7 (WORD) | 1.7(5.2) | 0.74 | 15.9(15.1) | 0.29 | 0.1(1.2) | 0.96 | 82.3(16.1) | 3x10^-7^ | 2x10^-5^(0.1) | 1.00 |
| VIQ 8 (WISC-III) | 0.4(2.3) | 0.84 | 33.0(20.0) | 0.10 | 0.1(1.6) | 0.95 | 66.4(19.9) | 8x10^-4^ | 5x10^-5^(0.1) | 1.00 |
| PIQ 8 (WISC-III) | 5.0(11.2) | 0.65 | 0.4(2.9) | 0.89 | 2.7(9.0) | 0.76 | 91.8(15.1) | 1x10^-9^ | 0.01(1.7) | 1.00 |

a. Proportion of genetic influences for expressive vocabulary at 15 months with respect to the total mid-childhood ability SNP-h^2^: a_51_*a_51_ / (a_51_*a_51_ + a_52_*a_52_ + a_53_*a_53_ + a_54_*a_54_ + a_55_*a_55_) * 100%

b. Proportion of genetic influences for expressive vocabulary at 24 months with respect to the total mid-childhood ability SNP-h^2^: a_52_*a_52_ / (a_51_*a_51_ + a_52_*a_52_ + a_53_*a_53_ + a_54_*a_54_ + a_55_*a_55_) * 100%

c. Proportion of genetic influences for expressive vocabulary at 38 months with respect to the total mid-childhood ability SNP-h^2^: a_53_*a_53_ / (a_51_*a_51_ + a_52_*a_52_ + a_53_*a_53_ + a_54_*a_54_ + a_55_*a_55_) * 100%

d. Proportion of genetic influences for receptive vocabulary at 38 months with respect to the total mid-childhood ability SNP-h^2^: a_54_*a_54_ / (a_51_*a_51_ + a_52_*a_52_ + a_53_*a_53_ + a_54_*a_54_ + a_55_*a_55_) * 100%

e. Proportion of genetic influences for mid-childhood LRA with respect to the total mid-childhood ability SNP-h^2^: a_55_*a_55_ / (a_51_*a_51_ + a_52_*a_52_ + a_53_*a_53_ + a_54_*a_54_ + a_55_*a_55_) * 100%

Factorial co-heritability reflects the proportion of the total SNP-h^2^ estimated for a mid-childhood ability explained by a specific genetic factor. SEs were derived using the Delta method and *P*-values based on a Wald-test assuming normality (S4 Text).

Abbreviations: a, accuracy; c, comprehension; LRA, language- and literacy-related ability; PIQ, performance intelligence quotient; VIQ, verbal intelligence quotient; voc, vocabulary; WORD, WISC-III, Wechsler Intelligence Scale for Children III
