# Supplementary material for: The developmental genetic architecture of vocabulary skills during the first three years of life: Capturing emerging associations with later-life reading and cognition
Source: PLoS Genet. 2021 Feb 12;17(2):e1009144. doi: 10.1371/journal.pgen.1009144 (PMC7880480; doi:10.1371/journal.pgen.1009144)
Supplement: S11 Table — (DOCX) [file pgen.1009144.s016.docx]

**S11 Table. Bivariate heritability for early-life vocabulary measures and mid-childhood reading, verbal intelligence and performance intelligence**

| **Measure** | **Bivariate heritability** | | | | | | | |
| --- | --- | --- | --- | --- | --- | --- | --- | --- |
|  | **Expressive voc**  **15 months** | | **Expressive voc**  **24 months** | | **Expressive voc**  **38 months** | | **Receptive voc**  **38 months** | |
|  | **Estimate (SE)** | ***P*** | **Estimate (SE)** | ***P*** | **Estimate (SE)** | ***P*** | **Estimate (SE)** | ***P*** |
| Reading a/c 7 (WORD) | -0.24(0.34) | 0.48 | 0.29(0.20) | 0.13 | 0.36(0.25) | 0.15 | 0.87(0.21) | 3x10^-5^ |
| VIQ 8 (WISC-III) | -0.19(0.49) | 0.69 | 0.54(0.19) | 0.004 | 0.60(0.24) | 0.01 | 0.88(0.16) | 8x10^-8^ |
| PIQ 8 (WISC-III) | -0.88(0.94) | 0.35 | -0.12(0.36) | 0.74 | 0.17(0.44) | 0.70 | 0.68(0.27) | 0.01 |

Bivariate heritability, reflecting the proportion of the phenotypic covariance that is accounted for by the genetic covariance. Standard errors (SEs) were approximated by the SE of the genetic covariance divided by the phenotypic covariance (as the SE of the phenotypic covariance is small) and *P*-values are based on a Wald-test, assuming normality (S5 Text).

Abbreviations: a, accuracy; c, comprehension; PIQ, performance intelligence quotient; VIQ, verbal intelligence quotient; voc, vocabulary; WORD, WISC-III, Wechsler Intelligence Scale for Children III
