# Supplementary figures and images for: The developmental genetic architecture of vocabulary skills during the first three years of life: Capturing emerging associations with later-life reading and cognition
Source: PLoS Genet. 2021 Feb 12;17(2):e1009144. doi: 10.1371/journal.pgen.1009144 (PMC7880480; doi:10.1371/journal.pgen.1009144)

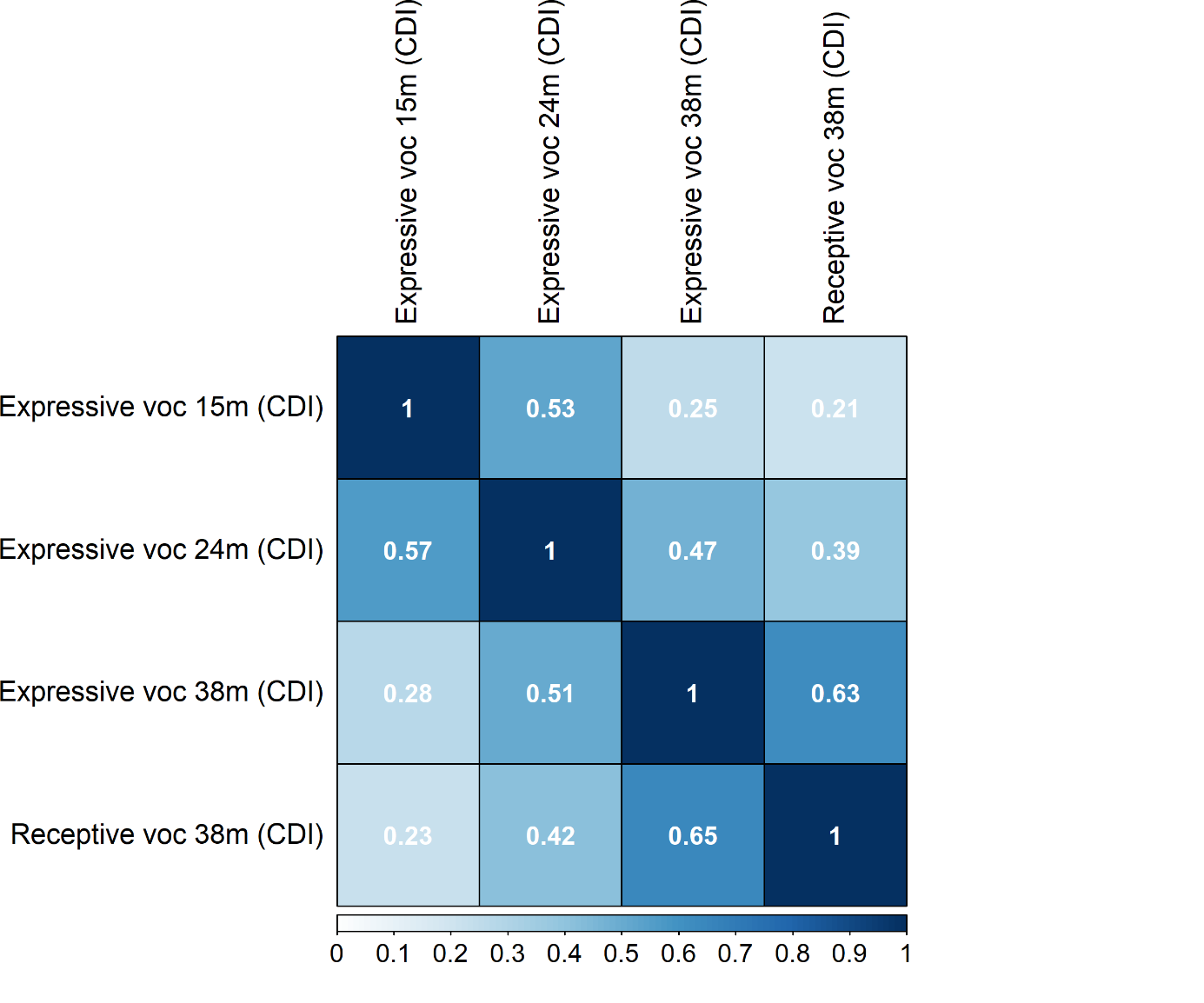

Supplement: S1 Fig — Phenotypic correlations among untransformed (lower triangle) and rank-transformed (upper triangle) measures with sufficient evidence for SNP-h2 (P>0.05) were estimated with Spearman’s rank and Pearson correlation coefficients respectively. All phenotypic correlation coefficients passed the significance threshold of P<0.05. Abbreviations: CDI, Communicative Development Inventory; m, months; voc, vocabulary. (TIF) [file pgen.1009144.s017.tif]

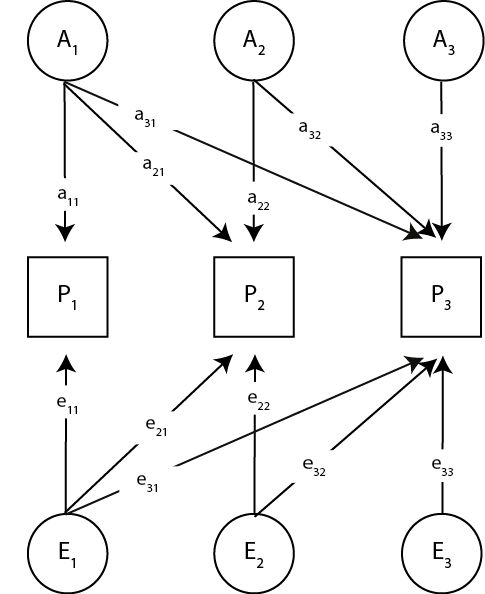

Supplement: S2 Fig — The variance/covariance structure of multivariate trait consisting of three standardised measures P1, P2 and P3 can be described using a Cholesky decomposition consisting of three genetic factors (A1, A2 and A3) and three residual factors (E1, E2 and E3), shown here with genetic and residual factor loadings (path coefficients). The observed phenotypic measures are represented by squares, while all latent genetic and residual factors are represented by a circle. Single headed arrows (’paths’) denote causal relationships between variables and are shown for genetic factor loadings (a) and residual factor loadings (e). Note that the variance of latent variables is constrained to unit variance, this is omitted from the diagrams to improve clarity. (TIF) [file pgen.1009144.s018.tif]
